# Bias in Mendelian randomization due to assortative mating

**DOI:** 10.1002/gepi.22138

**Published:** 2018-07-03

**Authors:** Fernando Pires Hartwig, Neil Martin Davies, George Davey Smith

**Affiliations:** ^1^ Postgraduate Programme in Epidemiology Federal University of Pelotas Pelotas Brazil; ^2^ Medical Research Council Integrative Epidemiology Unit University of Bristol Bristol UK; ^3^ Population Health Sciences Bristol Medical School University of Bristol Barley House Oakfield Grove Bristol UK

**Keywords:** ALSPAC, assortative mating, bias, causal inference, Mendelian randomization

## Abstract

Mendelian randomization (MR) has been increasingly used to strengthen causal inference in observational epidemiology. Methodological developments in the field allow detecting and/or adjusting for different potential sources of bias, mainly bias due to horizontal pleiotropy (or “off‐target” genetic effects). Another potential source of bias is nonrandom matching between spouses (i.e., assortative mating). In this study, we performed simulations to investigate the bias caused in MR by assortative mating. We found that bias can arise due to either cross‐trait assortative mating (i.e., assortment on two phenotypes, such as highly educated women selecting taller men) or single‐trait assortative mating (i.e., assortment on a single phenotype), even if the exposure and outcome phenotypes are not the phenotypes under assortment. The simulations also indicate that bias due to assortative mating accumulates over generations and that MR methods robust to horizontal pleiotropy are also affected by this bias. Finally, we show that genetic data from mother–father–offspring trios can be used to detect and correct for this bias.

## 1 INTRODUCTION

Genetic associations have been increasingly used in epidemiology to strengthen causal inferences regarding the association between a modifiable exposure and a given health outcome—a design termed Mendelian randomization (MR; Davey Smith & Ebrahim, 2003; Davey Smith & Hemani, 2014). Although MR studies are observational in nature, they are robust to several biases that can plague traditional observational studies. This is because the instruments (i.e., predictors of the exposure variable, such as a disease risk factor) are germline genetic variants, which are determined at conception and do not change throughout life. This eliminates reverse causation, where the (potentially preclinical) symptoms of disease affect the exposure. Moreover, given Mendel's first and second laws, germline genetic variants are unlikely to be associated with “classical” confounders (e.g., socio‐economic and lifestyle factors; Davey Smith et al., 2007) or with one another (except for variants in linkage disequilibrium; Davey Smith, 2011).

Valid causal inference using MR requires that the instrumental variable assumptions hold. This means that the genetic instrument must be associated with the exposure variable, and the association (if any) of the genetic instrument(s) with the outcome must be entirely mediated by the exposure (Davey Smith & Hemani, 2014). Although the first assumption is empirically verifiable, bias due to “off‐target” genetic effects (or, more formally, horizontal pleiotropy; Davey Smith & Hemani, 2014; Paaby & Rockman, 2013) can never be ruled out. Recently developed methods allow relaxation of this assumption in different ways (Bowden, Davey Smith, & Burgess, 2015; Bowden, Davey Smith, Haycock, & Burgess, 2016; Hartwig, Davey Smith, & Bowden, 2017), and are useful sensitivity analyses that may strengthen or weaken causal inference (Burgess, Bowden, Fall, Ingelsson, & Thompson, 2017). Other important sources of bias in MR include presence of population structure (such as ancestry‐related population stratification, family structure, and cryptic relatedness; Price, Zaitlen, Reich, & Patterson, 2010), linkage disequilibrium with one or more variants involved in other biological processes that influences the outcome (Davey Smith, & Ebrahim, 2003; Davey Smith, & Hemani, 2014), and selection bias, if genetic instruments influence the likelihood of participating in a study or of being followed‐up (Anderson et al., 2011; Munafo, Tilling, Taylor, Evans, & Davey Smith, 2018).

Although the biases above are widely recognized and have been the focus of methodological developments aimed at minimizing their influence, there are other potential sources of bias that could threaten causal inference. In this paper, we focus on assortative (nonrandom) mating, which occurs when people do not choose their partners at random, but rather based on particular characteristics (Jiang, Bolnick, & Kirkpatrick, 2013; Pearson, 1903). Assortative mating can be classified into single‐ or cross‐trait assortative mating. Single‐trait assortative mating occurs when individuals match on a particular trait, for example, tall women are more likely to select tall men (Tenesa, Rawlik, Navarro, & Canela‐Xandri, 2016). Cross‐trait assortative mating occurs when individuals of one trait are more likely to select individuals of another trait, for example, women with high intelligence test scores selecting taller men (Keller et al., 2013).

Our aim was to perform a simulation study to help clarify when assortative mating leads to bias in MR studies. We focused on genetically driven bias—that is, when assortment leads to a genetic correlation between parents (as explained in detail in the Methods). We also evaluate how methods robust to horizontal pleiotropy perform in the presence of assortative mating, and present approaches to detect and correct for this bias.

## 2 METHODS

### 2.1 Graphical representation of assortative mating

**Figure 1:**
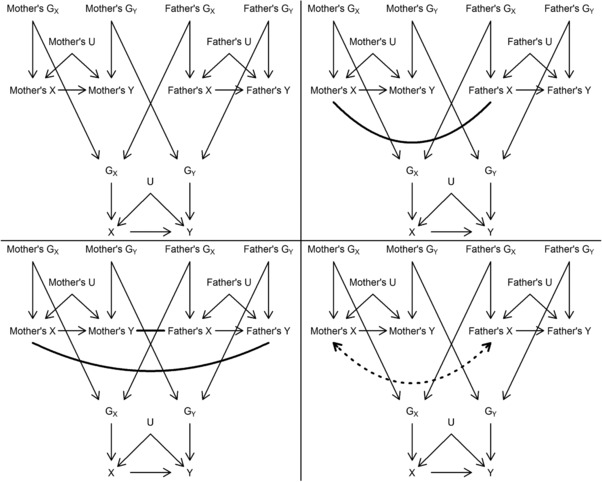
Causal diagrams depicting causal structures corresponding to mother–father–offspring trios and assortative mating *Note*. Top left panel: no assortment. Top right panel: representation of single‐trait assortment on *X* using a nondirected thick line. Bottom left panel: representation of cross‐trait assortment on *X* and Y using two nondirected thick lines. Bottom right panel: unsatisfactory representation of single‐trait assortment on *X* using a dotted bidirected arrow (which typically denote latent common causes). *X*: exposure phenotype; *Y*: outcome phenotype; *U*: unmeasured common cause of *X* and *Y*; $G_{X}$: collection of genetic variants with direct effects on *X*; $G_{Y}$: collection of genetic variants with direct effects on *Y*

Figure 1 illustrates why assortative mating may lead to bias in MR using causal diagrams. $G_{X}$ and $G_{Y}$ denote the genetic influences on the exposure (*X*) and outcome (*Y*) phenotypes. It is assumed that there is no horizontal pleiotropy between *X* and *Y*, so that all genetic variants that belong to $G_{X}$ are valid genetic instruments of *X*. This will facilitate detection of assortative mating bias using d‐separation rules. The collective effect of unmeasured common causes of *X* and *Y* is represented by *U*. We will interpret the causal diagrams assuming faithfulness, according to which d‐connection (i.e., presence of at least one open path from one variable to another) implies statistical association.

The top left panel depicts a situation with no assortative mating. It can be seen that $G_{X}$ is only related to *Y* via the path $G_{X}\to X\to Y$. Therefore, $G_{X}$ will associate with *Y* only if the path X→Y exists (i.e., if *X* has a causal effect on *Y*), and therefore is a valid instrument to assess the causal effect of *X* on *Y*.

The top right panel depicts a situation with single‐trait assortative mating (indicated by the thick line) on *X*. We used nondirected lines rather than arrows to denote assortative mating. Single‐trait assortative mating induces an association between mother's $G_{X}$ and father's $G_{X}$. This is because if people with high values of *X* tend to select partners with high values of *X* (i.e., positive assortment on *X*), then, by consequence of assortment at the phenotypic level, mother–father pairs will also be positively genetically correlated for *X*. However, the association of mother's *X* and father's *X* does not open any backdoor paths between $G_{X}$ and *Y*, so $G_{X}$ is a valid genetic instrument even in the presence of single‐trait assortative mating on *X*. The same reasoning applies to single‐trait assortative mating on *Y*. However, in some situations single‐trait assortative mating can render $G_{X}$ and *Y* associated in the absence of a causal effect of *X* on *Y*, as we show next using simulations.

The bottom‐left panel illustrates cross‐trait assortative mating on *X* and *Y* by two thick lines: from mother's *X* to father's *Y*, and from mother's *Y* to father's *X*. In this situation, if *X* does not cause *Y*, the mother's $G_{X}$ and father's $G_{Y}$, and mother's $G_{Y}$ and father's $G_{X}$ will be associated. If *X* does cause *Y*, then all parental genetic variables will associate. Therefore, cross‐trait assortative mating can induce associations between $G_{X}$ and *Y* even in the absence of a causal effect of *X* on *Y*. This invalidates the MR assumptions.

The bottom right panel provides a justification for representing assortment using nondirected lines rather than bidirected arrows (which typically represent latent common causes). The path mother's $G_{X}\to$ mother's X **↔** father's X← father's $G_{X}$ is not open, because both mother's *X* and father's *X* are colliders (i.e., each has two arrows pointing at it) on the path. Therefore, mother's $G_{X}$ and father's $G_{X}$ are not associated in this graph. Therefore, attempting to graphically represent assortment using bidirected arrows between parents’ phenotypes would imply in saying that assortment at the phenotypic level does not result in a genetic correlation between spouses, which is implausible.

### 2.2 Using parent's genetic data to control for assortative mating bias

The bottom left panel in Figure 1 shows that cross‐trait assortative mating on *X* and *Y* induces associations between $G_{X}$ and *Y*. This means $G_{X}$ is an invalid instrument to assess the causal effect of *X* on *Y*, leading to bias in MR analyses. However, this bias can be counteracted by conditioning on measured variables that block such bias sources (without creating new ones).

Figure 1 (bottom left panel) suggests that conditioning on $G_{Y}$ would control for bias due to assortative mating, since all open backdoor paths from $G_{X}$ to *Y* are mediated by $G_{Y}$, and conditioning on $G_{Y}\;$does not create any new open backdoor path. However, it is important to remember that $G_{Y}$ represents all genetic influences on $G_{Y}$, not all of which will be known. This can be seen more clearly in Supporting Information Figure 1, which shows two nonoverlapping sets of genetic influences on each phenotype: $G_{X}^{M}$ and $G_{X}^{U}$ represent measured and unmeasured genetic influences on *X*, respectively, such that $G_{X}=\left\{G_{X}^{M},G_{X}^{U}\right\}\;$; $G_{Y}^{M}$ and $G_{Y}^{U}$ are defined analogously, with respect to *Y*. Conditioning on the measured genotypes ($G_{Y}^{M}$) does not control for unmeasured genetic differences ($G_{Y}^{U}$). Therefore, unless all genetic influences on *Y* are known, measured and properly modelled, adjustment for genetic influences on *Y* may mitigate, but it is unlikely to eliminate, assortative mating bias.

An alternative way to overcome this bias is to adjust for parental genotype. Figure 1 (bottom left panel) illustrates that conditioning on mother's and father's $G_{X}$ block the open backdoor paths between $G_{X}$ and *Y* without creating new ones. Supporting Information Figure 1 shows that this also holds even if only a subset of all genetic influences on *X* are measured—that is, conditioning on mother's and father's $G_{X}^{M}$ blocks all backdoor paths (due to assortative mating) between $G_{X}^{M}$ and *Y*. Given that genetic instruments of *X* are a subset of $G_{X}$, this approach can be used to control for assortative mating bias without measuring all genetic influences on *X*, and requires measuring the genetic instruments in individuals (to assess the causal effect of *X* on *Y*) and their parents (to adjust for bias).

### 2.3 Simulation study

We performed a series of simulations to evaluate bias due to assortative mating in MR. The main goals of the simulation study were to (a) demonstrate that cross‐trait assortative mating on *X* and *Y* leads to bias in MR and (b) assess the strategy of using parent's $G_{X}^{M}$ to control for assortative mating bias. Additional simulations (described in the Supporting Information Methods and illustrated in Supporting Information Figure 2) were performed to explore additional scenarios of cross‐trait mating, and to demonstrate that in some situations, single‐trait assortative mating may also bias MR.

A detailed description of the simulation model is provided in the Supporting Information Methods. We simulated mother–father–offspring trios as depicted in Figure 1. Results were averaged across 5,000 simulated datasets. All simulated genetic variants were single nucleotide polymorphisms (SNPs). In each scenario, 40,000 trios, 50 SNPs with direct effects on *X* (corresponding to $G_{X}$ in Figure 1), and 50 SNPs (unless stated otherwise) with direct effects on *Y* (corresponding to $G_{Y}$ in Figure 1) were simulated. If ${G_{X}}_{k}$ denotes the *k*th genetic variant with a direct effect on *X*, then $G_{X}={\left\{{G_{X}\;}_{k}\right\}}_{k=1}^{50}$; the set of all genetic variants with direct effects on *Y* can be analogously defined as $G_{Y}={\left\{{G_{Y}}_{k}\right\}}_{k=1}^{50}$. All variants in the $G_{X}$ set have linear and additive effects on *X*; since $G_{X}$ corresponds to the entire genetic component of *X*, the amount of variance in *X* explained by $G_{X}$ is the narrow‐sense heritability of *X* ($h_{X}^{2}$), which equals the broad‐sense heritability of *X* (due to the absence of nonadditive components of genetic variance) in our simulations. In scenarios where *X* has no causal effect on *Y*, the same interpretation holds for $h_{Y}^{2}$ with respect to *Y*.

All genetic variants in $G_{X}$ were combined into an additive allele score $Z_{X}$, and the direct effect of $Z_{X}$ on *X* was controlled by the $\delta_{X}$ parameter; $Z_{Y}$ and $\delta_{Y}$ are defined analogously with respect to *Y* (see the Supporting Information Methods for details). Therefore, hX2=(δX2 var (ZX))/ var (X) and (again assuming no causal effect of *X* on *Y*) hY2=(δY2 var (ZY))/ var (Y). Given that in our model assortative mating model leads to changes in genetic and phenotypic variances while $\delta_{X}$ and $\delta_{Y}$ are structural parameters, the actual values of $h_{X}^{2}$ and $h_{Y}^{2}$ were higher in simulations with positive assortment than in simulations without assortment when keeping $\delta_{X}$ and $\delta_{Y}$ constant. However, in our simulations such differences were very small, so for simplicity we will ignore that $h_{X}^{2}$ and $h_{Y}^{2}$ are affected by assortative mating when presenting and discussing the results.

Positive assortment, which leads to a positive correlation between parents, was simulated using proxies of the phenotypes of interest, so as to allow control over the strength of the assortment. Cross‐trait assortative mating on *X* and *Y* was induced by pairing women and men according to $X^{P}$ and $Y^{P}$ (therefore, the correlation between spouses for $X^{P}$ and $Y^{P}$ is 1). These variables were equal to *X* and *Y*, respectively, plus random error terms, such that cor(*X*,$X^{P})=\mathrm{cor}(Y$,$Y^{P})=P\;\in \left[0,1\right]$. Small values of *P* imply that people weakly assort on *X* and *Y*, whereas high values of *P* imply that they strongly assort on *X* and *Y*. All other factors influencing partnering preferences are embedded in the error terms.

To mimic a bidirectional process, we initially paired women and men at random (so as to not induce assortment), and randomly divided the resulting women–men pairs into two sets. In one set of women–men pairs, men were sorted in ascending order of $Y^{P}\;$, and women were sorted in ascending order of $X^{P}$; in the other set of women–men pairs, men were sorted in ascending order of $X^{P}$, and women were sorted in ascending order of $Y^{P}$. The two sorted sets of women–men pairs were then combined together, preserving the order resulting from the sorting steps above, generating the full dataset of mother–father pairs.

We also simulated scenarios with no assortative mating and a nonzero causal effect of *X* on *Y*. This scenario was used to evaluate the performance of selected MR methods to detect a causal effect.

### 2.4 Statistical analyses

We investigated the bias and the coverage of different MR estimators across these scenarios. The causal effect of *X* on *Y* was estimated using two‐stage least squares regression (TSLS). Unless stated otherwise, all 50 genetic variants in $G_{X}$ were combined in an weighted additive allele score, which was used as the instrumental variable (IV) for *X*. The weights ($\omega ={\left\{\omega_{k}\right\}}_{k=1}^{50}$) were obtained by regressing *X* on each genetic variant in $G_{X}$ in one random half of the simulated dataset. To avoid overfitting, those weights were used to construct the IV in the other half of the data.

Three versions of the TSLS method were performed:

- TSLS (1): estimating the causal effect of the exposure on the outcome with no covariates. The causal effect estimate was obtained by fitting the following two linear regression models:
  $$\;{\hat{X}}_{i}={\hat{\beta }}_{0}+{\hat{\beta }}_{1}\times S_{i},$$

where $\hat{X}$ is the value of *X* predicted by the model (this is because the error term is omitted), ${\hat{\beta }}_{0}$ is the intercept estimate, and ${\hat{\beta }}_{1}$ is the estimate of the change in *X* associated with a unit increment in *S* (which is the individual's allele score—i.e., the IV).

$$\;{\hat{Y}}_{i}={\hat{\gamma }}_{0}+{\hat{\gamma }}_{1}\times {\hat{X}}_{i},$$

where $\hat{Y}$ is the value of *Y* predicted by the model (this is because the error term is omitted), ${\hat{\gamma }}_{0}$ is the intercept estimate, and ${\hat{\gamma }}_{1}$ is the estimate of the change in *Y* associated with a unit increment in $\hat{X}$—that is, the estimate of the causal effect of the exposure *X* on the outcome *Y*.

- TSLS (2): estimating the causal effect of the exposure on the outcome adjusting for parental allele scores. The causal effect estimate was obtained by fitting the following two linear regression models:
  $$\;{\hat{X}}_{i}^{*}={\hat{\beta }}_{0}^{*}+{\hat{\beta }}_{1}^{*}\times S_{i}+{\hat{\beta }}_{2}^{*}\times S_{i}^{m}+{\hat{\beta }}_{3}^{*}\times S_{i}^{f},$$

where $S^{m}$ and $S^{f}$ denote mother's and father's respective allele scores.

$$\;{\hat{Y}}_{i}^{*}={\hat{\gamma }}_{0}^{*}+{\hat{\gamma }}_{1}^{*}\times {\hat{X}}_{i}^{*}+{\hat{\gamma }}_{2}^{*}\times S_{i}^{m}+{\hat{\gamma }}_{3}^{*}\times S_{i}^{f},$$

where ${\hat{\gamma }}_{1}^{*}$ is the estimate of the causal effect of the exposure $X^{*}$ on the outcome $Y^{*}$.

For the next method, we constructed allele scores using nontransmitted alleles (Lawlor et al., 2017; Zhang et al., 2015)—that is, the parents’ alleles that were not transmitted to the offspring. For example, consider that the mother's genotype for a given genetic variant is AT, and that the offspring's genotype for the same genetic variant is AA. By comparing mother's and offspring's genotypes, it can be seen that the mother transmitted the A, and not the T allele, to the offspring. The same applies for parent's nontransmitted alleles. In our application, mother's and father's nontransmitted allele scores were determined for all genetic variants used to compute the IV, and were used to compute nontransmitted weighted (using the same weights described above) allele scores by the same procedure used to compute regular weighted allele scores.

- TSLS (3): jointly estimating the causal effect of the exposure on the outcome, and the direct effects of parent's exposure phenotypes on (offspring's) outcome using paternal nontransmitted allele scores as instruments of parent's exposure phenotype; therefore, this analysis has three exposure variables: offspring's, mother's, and father's exposure phenotypes, three IVs: offspring's allele score, mother's nontransmitted allele score, and father's nontransmitted allele score, respectively, and one outcome variable: offspring's outcome phenotype. The causal effect estimate was obtained by fitting the following four linear regression models:
  $$\;{{\hat{X}}_{i}}^{\prime }={{\hat{\beta }}_{0}}^{\prime }\;+{\hat{\beta }}_{1}^{\prime }\times S_{i}+{\hat{\beta }}_{2}^{\prime }\times {W_{i}}^{m}+{\hat{\beta }}_{3}^{\prime }\times {W_{i}}^{f},$$
  $${\hat{X}}_{i}^{m\prime }={\hat{\beta }}_{0}^{m^{\prime }}+{\hat{\beta }}_{1}^{m^{\prime }}\times S_{i}+{\hat{\beta }}_{2}^{m^{\prime }}\times {W_{i}}^{m}+{\hat{\beta }}_{3}^{m^{\prime }}\times {W_{i}}^{f},$$
  $${\hat{X}}_{i}^{f^{\prime }}={\hat{\beta }}_{0}^{f^{\prime }}+{{\hat{\beta }}_{1}}^{f^{\prime }}\times S_{i}+{\hat{\beta }}_{2}^{f^{\prime }}\times {W_{i}}^{f}+{\hat{\beta }}_{3}^{f^{\prime }}\times {W_{i}}^{f},$$

where $W^{m}$ and $W^{f}$ denote mother's and father's, respectively, nontransmitted allele scores, and ${\hat{X}}^{m^{\prime }}$ and ${\hat{X}}^{f^{\prime }}$ denote mother's and father's, respectively, predicted exposure phenotype.

$${\hat{Y}}_{i}^{\prime }={\hat{\gamma }}_{0}^{\prime }\;+{\hat{\gamma }}_{1}^{\prime }\times {\hat{X}}_{i}^{\prime }+{\hat{\gamma }}_{2}^{\prime }\times {\hat{X}}_{i}^{m^{\prime }}+{\hat{\gamma }}_{3}^{\prime }\times {\hat{X}}_{i}^{f^{\prime }},$$

where ${\hat{\gamma }}_{1}^{\prime }$ is the estimate of the causal effect of the individual's exposure $X^{\prime }$ on the individual's outcome $Y^{\prime }$, ${\hat{\gamma }}_{2}^{\prime }$ is an estimate of the direct effect of mother's exposure $X^{m^{\prime }}$ on the individual's outcome $Y^{\prime }$, and ${\hat{\gamma }}_{3}^{\prime }$ is an estimate of the direct effect of father's exposure ${X^{f}}^{\prime }$ on the individual's outcome $Y^{\prime }$.

TSLS (2) and TSLS (3) aim at providing both a causal effect estimate that is robust to assortative mating and a test of whether or not assortative mating bias is present. Of note, the equations above are for explanation only, since the estimates were based on TSLS (as mentioned above), which takes account of the estimation error in the first‐stage (i.e., in the prediction of the exposure phenotype).

For each of those methods, the causal effect estimate and false rejection rate of the 95% confidence intervals were calculated. Additional analyses (described in the Supporting Information Methods) were performed to evaluate the performance of summary data MR methods robust to horizontal pleiotropy, and of tests commonly used to detect horizontal pleiotropy in the summary data setting.

### 2.5 Empirical example: Height and education using Avon Longitudinal Study of Parents and Children

Previous studies have used MR in samples of unrelated individuals to investigate the causal effect of height on educational attainment (Tyrrell et al., 2016). If parents assort on height and education or if there are dynastic effects of parental height or education on their offsprings’ outcomes, then MR may suffer from bias. We evaluated this using data from the Avon Longitudinal Study of Parents and Children (ALSPAC). ALSPAC sampled 14,541 pregnant women between April 1, 1991 and December 31, 1992. Full details of the study have been published elsewhere (Boyd et al., 2013; Fraser et al., 2013).

The study participants have been followed up for almost 30 years, and the mothers, partners, and offspring have completed questionnaires and the offspring have had their educational records linked from the National Pupil Database. Ethical approval for the study was obtained from the ALSPAC Ethics and Law Committee and the Local Research Ethics Committees. Please note that the study website contains details of all the data that are available through a fully searchable data dictionary. The mothers, fathers, and offspring have also provided biological samples for genotyping. We extracted the 691 of the 697 genetic variants known to associate with height, respectively, at *P* < 5 × 10^−8^ (Wood et al., 2014). We defined offspring educational attainment using average points scored in GCSE examinations taken at age 16. Offspring height was measured during their clinic visit at age 17.5. We estimated the correlations between mother, father, and offspring phenotypes and genotypes. We estimated the effect of height on educational attainment using the height allele score as an IV using the offspring data alone (i.e., TSLS (1)), and additionally adjusting for parental allele scores (i.e., TSLS (2)).

Scripts used to perform the simulations and to analyze ALSPAC data are available at: https://github.com/FernandoHartwig/AssortativeMating_Scripts.

## 3 RESULTS

### 3.1 Simulation study

**Figure 2:**
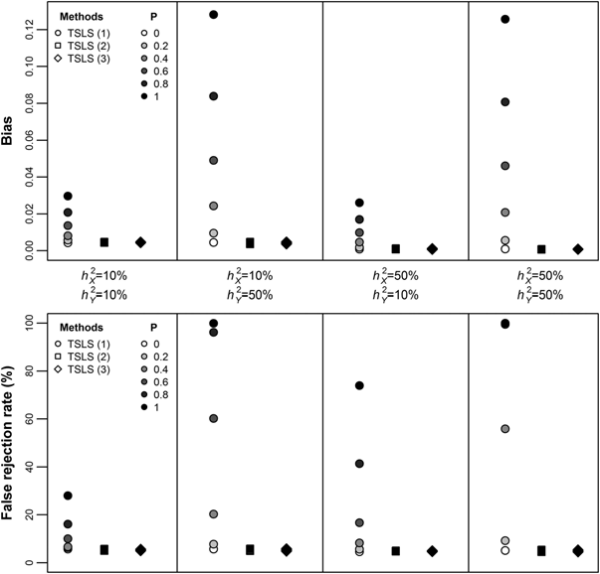
Bias and false‐rejection rates of two‐stage least squares (TSLS) regression methods in the presence of cross‐trait assortative mating on *X* and *Y* under no causal effect of *X* on *Y* for different levels of assortment (*P*) and narrow‐sense heritability of *X* ($h_{X}^{2}$) and *Y* ($h_{Y}^{2}$) *Note*. TSLS (1): no covariates; TSLS (2): adjusting for parental allele scores; TSLS (3): joint estimation of parental and individual's effects, using parental nontransmitted allele scores as instruments of parental phenotype

Figure 2 shows that TSLS is positively biased when there is positive cross‐trait assortative mating on *X* and *Y*. The bias increased proportionally with increasing the degree of assortment. However, both TSLS (2) (i.e., adjusting for parent's allele scores) and TSLS (3) (i.e., jointly modelling individual's and parental effects, using nontransmitted allele scores as instruments of parental phenotype) were unbiased with false discovery rates close to 5%. Figure 2 also indicates that the bias was much stronger when the outcome *Y* was highly heritable, while changing the heritability of *X* had virtually no effect on bias (although it influences power because it affects instrument strength, and therefore influences weak instrument bias—although the latter was purposely negligible in our simulations to isolate bias due to assortment as much as possible). The bias was also invariant to whether all or subset of variants in $G_{X}$ are used to construct the IV and to the total number of variants in $G_{Y}$, thus corroborating the notion that the bias depends mainly on the degree of assortment and heritability of *Y* (Table 1). Moreover, TSLS (2) and TSLS (3) were unbiased regardless of whether all or a subset of variants in $G_{X}$ are used to construct the IV, as long as parental allele scores include the same variants with the same weights as the IV, thus corroborating the notion that our approach requires only that the genetic instruments (rather than all variants in $G_{X}$) are measured in study individuals and their parents.

**Table 1:** Bias and standard error (*SE*) of the conventional two‐stage least squares (TSLS(1)) regression in the presence of cross‐trait assortative mating on *X* and *Y* under no causal effect of *X* on *Y* and high narrow‐sense heritability of *X* ( $h_{X}^{2}=\;0.5$), for different values of assortment strength (*P*), narrow‐sense heritability of *Y* ($h_{Y}^{2}$), number of genetic variants in $G_{X}$ used to calculate the genetic instrument (GI) of *X*, and for number of genetic variants in $G_{Y}$

			Number of variants in the GI of *X*			
			10^a^		50	
*P*	$h_{Y}^{2}$ (%)	Number of variants in $G_{Y}$	Bias	*SE*	Bias	SE
0.2	10	10	0.002	0.023	0.002	0.010
		50	0.002	0.023	0.002	0.010
	50	10	0.005	0.023	0.006	0.010
		50	0.005	0.023	0.006	0.010
0.6	10	10	0.010	0.023	0.010	0.010
		50	0.010	0.023	0.010	0.010
	50	10	0.046	0.022	0.046	0.010
		50	0.046	0.022	0.046	0.010
1.0	10	10	0.026	0.022	0.026	0.010
		50	0.026	0.022	0.026	0.010
	50	10	0.125	0.021	0.125	0.010
		50	0.126	0.021	0.126	0.010

a Randomly sampled from the entire set $G_{X}$ of 50 genetic variants with direct effects on *X*.

$G_{Y}$: set of all genetic variants with direct effects on *Y*.

**Figure 3:**
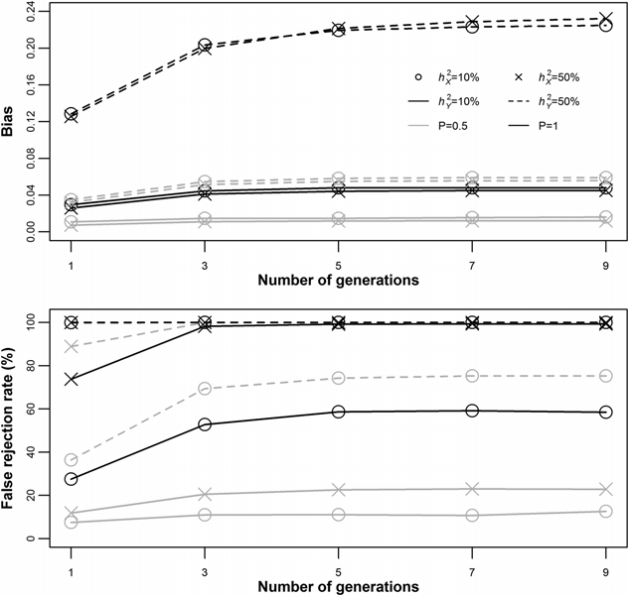
Bias and false‐rejection rates of the conventional two‐stage least squares regression (TSLS) method in the presence of cross‐trait assortative mating on *X* and *Y* over many generations under no causal effect of *X* on *Y* for different levels of assortment (*P*) and narrow‐sense heritability of *X* ($h_{X}^{2}$) and *Y* ($h_{Y}^{2}$)

Figure 3 shows that bias due to cross‐trait assortative mating on *X* and *Y* accumulates over generations, with the increment in bias from one generation to the next getting smaller for larger numbers of generations. Again, the TSLS (2) and TSLS (3) were unbiased, regardless of the number of generations (Supporting Information Table 1). However, the TSLS (2) and TSLS (3) methods have considerably lower power than the conventional TSLS (1) (Table 2).

**Table 2:** Performance of variations of the two‐stage least squares (TSLS) regression method to detect a causal effect of *X* on *Y* of 0.05 in absence of assortative mating

Parameters	Method	Estimate	Power (%)
$h_{X}^{2}=10\%$	TSLS (1)	0.054	69.8
$h_{Y}^{2}=10\%$	TSLS (2)	0.054	42.4
	TSLS (3)	0.054	42.3
$h_{X}^{2}=10\%$	TSLS (1)	0.055	69.8
$h_{Y}^{2}=50\%$	TSLS (2)	0.054	41.6
	TSLS (3)	0.054	41.6
$h_{X}^{2}=50\%$	TSLS (1)	0.053	91.1
$h_{Y}^{2}=10\%$	TSLS (2)	0.052	66.3
	TSLS (3)	0.052	66.2
$h_{X}^{2}=50\%$	TSLS (1)	0.053	91.3
$h_{Y}^{2}=50\%$	TSLS (2)	0.052	66.3
	TSLS (3)	0.052	66.2

TSLS (1): no covariates; TSLS (2): adjusting for parental allele scores; TSLS (3): adjusting for parental non‐transmitted allele scores; $h_{X}^{2}$: narrow‐sense heritability of *X*; $h_{Y}^{2}$: narrow‐sense heritability of *Y*.

Additional assortative mating patterns were also explored (see the Supporting Information Methods for a full description). Supporting Information Table 2 shows that cross‐trait assortative mating on variables other than *X* or *Y* can also lead to bias, as long the variables under assortment are genetically correlated (either through vertical or horizontal pleiotropy) with *X* and *Y*. Supporting Information Table 3 displays that some patterns of single‐trait assortative mating lead to bias in MR estimates. For this to happen, the variable under assortment must be genetically correlated with both *X* and *Y*, either through horizontal or through vertical pleiotropy. If *X* and *Y* are not genetically correlated, then single‐trait assortative mating does not bias MR (Supporting Information Table 4).

In analyses including summary data MR methods, it was observed that those methods were similarly biased to one another and to the conventional TSLS method, with false rejection rates varying according to the precision of each method (Supporting Information Figure 3 and Supporting Information Tables 2 and 3). Moreover, Supporting Information Table 5 illustrates that tests commonly used to detect horizontal pleiotropy in the two‐sample setting did not detect bias due to assortative mating in our simulations. However, parental genetic data can be used to detect this bias: both conventional allele scores (TSLS (2)) and nontransmitted allele scores (TSLS (3) and (4)) can be used for this purpose. Parental allele scores from TSLS (2) provide a test for the presence and direction of this bias, which was more powerful than the approaches based on nontransmitted allele scores. In the absence of assortative mating, all TSLS‐based tests for assortative mating had a false rejection close to 5%.

### 3.2 Illustrative example

We applied these methods to investigate the effect of height on educational attainment using a sample from ALSPAC. In total, 1,170 participants had phenotype and genotype data for mother, father, and offspring (summary statistics shown in Supporting Information Table 6). Mother and father's heights and education attainment phenotypes were correlated (Pearson correlation coefficients of 0.24 and 0.47, respectively). The mother and father's allele scores for height and education were more weakly correlated (Pearson correlation coefficients of 0.07 and 0.04, respectively) (Table 3). Linear regression suggested each additional 1 cm of height was associated with 0.031 (95% CI: [0.01, 0.07]) additional years of education (Table 4). The conventional MR estimates using TSLS (1) suggested that each 1 cm of height increased educational attainment by an additional 0.16 (95% CI: [0.07, 0.40]) years. After adjustment for parental allele scores for height (TSLS (2)), these estimates attenuated to 0.00 (95% CI: [−0.45, 0.45]).

**Table 3:** Phenotypic and genotypic correlations of height and education in ALSPAC mother–father offspring trios

		Height			Educational attainment		
		Mother	Father	Offspring	Mother	Father	Offspring
Phenotypic							
Height	Mother	1					
		*N* = 1113					
	Father	0.24^a^	1				
		*N* = 977	*N* = 989				
	Offspring	0.44^a^	0.36^a^	1			
		*N* = 1113	*N* = 989	*N* = 1170			
Education	Mother	0.10^a^	0.12^a^	0.01	1		
		*N* = 1109	*N* = 988	*N* = 1127	*N* = 1127		
	Father	0.08^a^	0.07^a^	0.05	0.47^a^	1	
		*N* = 1107	*N* = 987	*N* = 1125	*N* = 1125	*N* = 1125	
	Offspring	0.11^a^	0.09^a^	0.04	0.38^a^	0.32^a^	1
		*N* = 1113	*N* = 989	*N* = 1170	*N* = 1127	*N* = 1125	*N* = 1170
Genotypic (*N* = 1,170)							
Height	Mother	1					
	Father	0.07^a^	1				
	Offspring	0.53^a^	0.52^a^	1			
Education	Mother	−0.02	0.05	−0.02	1		
	Father	−0.01	−0.01	0.02	0.05	1	
	Offspring	−0.06^a^	−0.01	−0.03	0.55^a^	0.52^a^	1

ALSPAC: Avon Longitudinal Study of Parents and Children; *N*: sample size.

a *P *< 0.05.

**Table 4:** Changes of offspring academic attainment in years per 1 cm increase in height

			Confidence interval^a^		
Method	*N*	Mean difference	Lower	Upper	*P*‐value
Linear regression	1,170	0.060	−0.022	0.141	0.150
MR using TSLS (1)	1,170	0.162	−0.073	0.398	0.177
MR using TSLS (2)	1,170	0.000	−0.449	0.450	0.998

MR: Mendelian randomization; TSLS: two‐stage least squares regression; *N*: sample size; TSLS (1): no covariates; TSLS (2): adjusting for parental allele scores.

a Calculated using robust standard errors.

## 4 DISCUSSION

Our study characterized how assortative mating can induce bias in MR studies. Through causal diagrams and simulations covering a range of scenarios, we showed that this bias can occur when there is cross‐trait assortative mating on the exposure and outcome phenotypes, or on variables genetically correlated with them; or single‐trait assortative mating on a single phenotype genetically correlated with both the exposure and the outcome phenotypes (Table 5). Our simulations also indicated that the bias affects not only the conventional TSLS and inverse‐variance weighting (IVW) methods, but also the MR‐Egger regression (Bowden et al., 2015), weighted median (Bowden et al., 2016), and the mode‐based estimate (MBE) (Hartwig et al., 2017). These findings reenforce the point that those methods are not robust to all sources of bias, but only to some forms of horizontal pleiotropy.

**Table 5:** Bias in Mendelian randomization due to the investigated patterns of assortative mating

Trait(s) under assortment	Bias in MR
***Single‐trait assortative mating***	
Exposure phenotype	No
Outcome phenotype	No
Phenotype genetically correlated with both exposure and outcome via horizontal pleiotropy	Yes
Phenotype genetically correlated with both exposure and outcome via vertical pleiotropy	Yes
Exposure and outcome phenotypes	Yes
***Cross‐trait assortative mating***	
Exposure and outcome phenotypes	Yes
Phenotype genetically correlated with exposure and phenotype genetically correlated with outcome (both via horizontal pleiotropy)	Yes
Phenotype genetically correlated with exposure and phenotype genetically correlated with outcome (both via vertical pleiotropy)	Yes

This study evidenced that bias due to assortative mating is of greater concern when the strength of assortment is strong, when the outcome phenotype is highly heritable, and when the process has been going on over many generations. Many human phenotypes are suggested to have high heritability in the populations where they were studied (Speed, Cai, Johnson, Nejentsev, & Balding, 2017; Wang, Gaitsch, Poon, Cox, & Rzhetsky, 2017). In our simulations, cross‐trait assortative on *X* and *Y* mating resulted in realistic between‐parents correlations (Supporting Information Table 7). However, we know that most of this correlation is due to assortment, but in practice it can be challenging to differentiate phenotypic correlation within spouse pairs due to ethnically, geographically, and/or socially determined mating from assortative mating (Abdellaoui, Verweij, & Zietsch, 2014; Domingue, Fletcher, Conley, & Boardman, 2014b). Nuclear twin family models can potentially be used to detect assortative mating; for example, studies have reported evidence of positive cross‐trait assortative mating between height and intelligence (Keller et al., 2013). Another strategy would be to use data of genetic variant(s) known to associate with a given phenotype and test their association with a second phenotype between spouses. This strategy detected a positive association between a height allele score in women and education of their male spouses (Carslake D et al., 2015), as well as provided evidence for assortative mating involving height, educational attainment, and other phenotypes (Robinson et al., 2017). However, this strategy may be prone to other biases. For example, if the height allele score has horizontal pleiotropic effects on education, then single‐strait association involving height would result in correlation between maternal height and paternal education, and vice‐versa.

Recent studies using genetic data provided further insights into assortative mating in humans. For example, findings from a study in the U.K. Biobank were consistent with positive assortative mating for hypertension (or traits correlated with it, such as height), but the data were insufficient to differentiate between assortative mating and other potential sources of between‐spouses correlation (Munoz et al., 2016). Another study in the U.K. Biobank estimated that a person's own genotype (using ∼320,000 autosomal SNPs) accounts for 4.1% of the variability in the mate height choice, and that 89% of the genetic variation associated with a person's own height and mate height choice is shared. The same study also estimated that ∼5% of the height heritability is a result of assortative mating (Tenesa et al., 2016). In non‐Hispanic white participants in the Health and Retirement Study, spouses were genetically correlated, but such correlation was substantially weaker than the between‐spouse correlation regarding educational attainment. Moreover, genetic similarities between spouses explained only up to 10% of the correlation regarding education (Domingue, Fletcher, Conley, & Boardman, 2014a), suggesting that the environmental component of assortative mating on education is stronger than the genetic component, which would be expected given that such between‐spouse genetic correlations are a consequence of assortative mating at the phenotypic level.

Our simulations indicated that adjusting for parental allele scores is a simple and effective way to test and control for this bias. Nontransmitted allele scores can also be used, but they seemed to offer no advantage over the simple allele scores when the goal is to estimate the causal effect of the individual's exposure on the individual's outcome, which is the situation covered in our simulations. Nontransmitted allele scores have been proposed as genetic instrumental variables of maternal exposures on child's outcomes because they avoid the issue of horizontal pleiotropy due to effects of offspring's exposure on offspring's outcome (Lawlor et al., 2017; Zhang et al., 2015). Our findings indicate that nontransmitted allele scores also detect assortative mating bias; therefore, causal effect estimates of maternal exposures based on nontransmitted allele scores can be biased if the maternal exposure phenotype is under assortment, as previously noted by others (Lawlor et al., 2017; Zhang et al., 2015).

The MR with the direction of causation (MR‐DOC) twin model, which has been recently developed with the goal of testing for horizontal pleiotropy, could in principle be used to test and correct for assortative mating bias (Minica, Dolan, Boomsma, Geus, & Neale, 2018). Structural approaches to model, and thus correct for, bias sources in MR have been recently proposed. For example, structural equation modeling (SEM) can be used to estimate the causal effect of maternal exposures (Warrington, Freathy, Neale, & Evans, 2018), and should in principle be flexible enough to model assortative mating effects. In the case of MR‐DOC, a major disadvantage is the necessity of having twin data and that, in practice, some parameters of the model may have to be constrained. Our method is very simple, but requires trio data and is less flexible. It may also be possible to use methods that require less data.

Another possibility to mitigate bias due to assortative mating is to use outcome allele scores as covariates. However, our analyses using causal diagrams suggested that this approach is prone to residual bias unless all genetic variants that influence the outcome are measured and properly modelled. Nevertheless, it may be feasible to exploit genetic data on the outcome in other forms. For example, if SNPs in the exposure allele score are not in linkage disequilibrium with SNPs in the outcome allele score, then a nonzero correlation between exposure and outcome allele scores would be indicative of cross‐trait assortative mating (or some other phenomenon, such as population substructure). This could be exploited to detect and possibly correct for assortative mating bias, but further methodological work is required on this topic. Although comparing methods to detect and adjust for assortative mating will be useful, it is likely that the methods are complementary to each other, and choosing one over the other will depend on study‐specific factors such as the data available and the research question.

Although the notion that assortative mating can bias MR is widespread, this is the first study to thoroughly examine this issue in simulations, providing a quantitative assessment of the bias. For cross‐trait assortative mating, assuming that a plausible range of the correlation between mother's *X* and father's *Y* (and vice‐verse) is from 0.1 to 0.3, then (based on Supporting Information Table 7) plausible values of the assortment strength parameter *P* range, approximately, from 0.4 to 0.7. It is also plausible to assume that in general assortment has been occurring over many generations. Setting the number of generations to 9 (as in Figure 3), the bias ranged from 0.008 (for P=0.4) to 0.022 (for P=0.7) when setting the heritability of $Y(h_{Y}^{2})$ to 10%; when setting $h_{Y}^{2}$ = 50%, the bias ranged from 0.036 (for P=0.4) to 0.110 (for P=0.7). Given that  var (X)≈1 and  var (Y)≈1 in our simulations (as shown in Supporting Information Table 8), these bias estimates can be interpreted (approximately) as Pearson correlation coefficients. Importantly, those bias estimates are heavily dependent on our assumed data‐generating model. Therefore, extrapolating them to a practical situation requires parametric assumptions about the mechanism that generated the observed data.

One of the main strengths of our study was that we explored a variety of causal structures and assortment patterns, which allowed us to clarify when assortative mating is and is not likely to bias MR. In particular, we showed that even single‐trait assortative mating and assortment that is not directly on the exposure and outcome variable themselves can bias MR. We also showed that MR methods robust to horizontal pleiotropy are affected by this bias. Those conclusions were drawn using a data‐generating model that, while simple, presented characteristics expected under classical assortative mating models, such as increases in genetic and phenotypic variances (Supporting Information Table 8), as well as in the correlation between genetic variants (Supporting Information Table 9; Hedrick, 2017; Jorjani, Engström, Strandberg, & Liljedahl, 1997). However, it is important to note that this may not be a feature of all assortative mating models (Hedrick, 2017).

Any simulation model is a simplification of a likely much more complex reality. Our simulations were far from being an exhaustive list of all possible scenarios, implying that they do not illustrate some aspects of assortative mating bias. For example, when horizontal pleiotropic effects were simulated (in Scenarios 2 and 4), they were assigned to all genetic variants under consideration. This simplified the simulation model while allowing the main conclusions to be drawn. However, doing so prevented us from exploring more refined issues. For example, if some, but not all, of the genetic variants influence the variable(s) under assortment through horizontal pleiotropy, there will be between‐instrument heterogeneity, unlike in our simulations. This suggests that some of the robust MR methods, such as the median and the MBE, may be robust to assortative mating bias in those particular cases (provided that their assumptions hold). Therefore, it is possible that heterogeneity tests detect and some MR methods correct for assortative mating bias in some circumstances, but more firm conclusions require further methodological work. Therefore, our findings should be interpreted only as general indications on how assortative mating can influence MR, and extrapolating our conclusions to scenarios not covered in our simulations should be avoided.

We focused on how assortment on heritable phenotypes may lead to bias in MR by inducing a correlation between $G_{X}$ and $G_{Y}$. However, there are other forms that assortment can bias MR findings. For the sake of illustration, assume that intelligence is not heritable. Nevertheless, if more intelligent women tend to partner with taller men (and vice‐versa), a MR analysis assessing the causal effect of height on family earnings would be biased because partner's intelligence is likely to have a causal effect on family earnings. Further methodological development on how to detect and control for bias in cases such as this is required.

We demonstrated that there was little evidence of an effect of height on educational attainment after adjustment for parental genotype. This suggests that effects of height on educational attainment may be due to assortative mating or dynastic effects. In this sample, the biggest impact came from adjusting for father's allele score. However, our empirical results are imprecise and are provided for illustration. Future work should combine larger samples of related individuals to precisely estimate the effect of height on educational outcomes while controlling for assortative mating and dynastic effects.

It is possible in principle to combine the simple assortative mating bias adjustment approach presented here (i.e., include parental allele scores as covariates in the model) with methods that offer robustness to other biases, such as horizontal pleiotropy. For example, assortative mating bias adjustment could be combined with horizontal pleiotropy robust methods that require individual‐level data, such as Linear Slichter Regression (Spiller, Slichter, Bowden, & Davey Smith, 2017). It may even be possible to apply summary data methods (such as the ones we evaluated) to summary association results (i.e., instrument‐exposure and instrument‐outcome estimates and standard errors) for each genetic instrument, generated adjusting for assortative mating bias (e.g., using multivariable regression). Future methodological development is required to evaluate the theoretical and practical feasibility of those combinations, and to develop the best ways to do so. Combining methods robust to different bias sources in a single approach would be useful to obtain causal effect estimates robust to a range of biasing sources, which will strengthen causal inference using MR.

Our study reenforces assortative mating as a potential bias source in MR, and the utility of trio data to detect and adjust for this bias. Whenever possible, and especially when the phenotypes under consideration are likely to be under assortment, we recommend researchers to perform sensitivity analysis using trio data to test if assortative mating is present and, if so, to obtain causal effect estimates more robust to this bias.

## Supporting information

Supporting Information

Supporting Information

Supporting Information

Supporting Information
