# Supplementary material for: Bias in Mendelian randomization due to assortative mating
Source: Genet Epidemiol. 2018 Jul 3;42(7):608–20. doi: 10.1002/gepi.22138 (PMC6221130; doi:10.1002/gepi.22138)
Supplement: Supplementary file 1 — Supporting Information [file GEPI-42-608-s001.docx]

**Bias in Mendelian randomization due to assortative mating**

**Running Title:** Assortative mating in Mendelian randomization

Fernando Pires Hartwig^1,2*^, Neil Martin Davies^2,3^ and George Davey Smith^2,3^

^1^Postgraduate Programme in Epidemiology, Federal University of Pelotas, Pelotas, Brazil.

^2^Medical Research Council Integrative Epidemiology Unit, University of Bristol, BS8 2BN, United Kingdom.

^3^Population Health Sciences, Bristol Medical School, University of Bristol, Barley House, Oakfield Grove, Bristol, BS8 2BN, United Kingdom.

*Corresponding author. Postgraduate Program in Epidemiology, Federal University of Pelotas, Pelotas (Brazil) 96020-220. Phone: 55 53 981068670. E-mail: [fernandophartwig@gmail.com](mailto:fernandophartwig@gmail.com); [fh15144@bristol.ac.uk](mailto:fh15144@bristol.ac.uk).

**1 SUPPLEMENTARY METHODS**

**1.1 Simulation model**

For each individual in a mother-father-offspring trio (see Figure 1), we used an individual-level data-generating model for the $i$th individual described below:

The allele scores

$${Z_{X}}_{i}=\sum_{k=1}^{L_{X}} {\gamma_{X}}_{k}{G_{X}}_{ik} (1)$$

$${Z_{Y}}_{i}=\sum_{k=1}^{L_{Y}} {\gamma_{Y}}_{k}{G_{Y}}_{ik} (2)$$

, where ${G_{X}}_{k}$ is the $k$th genetic variant in $G_{X}$ (the set of all genetic variants with direct effects on $X$), and ${G_{Y}}_{k}$ is the $k$th genetic variant in $G_{Y}$ (the set of all genetic variants with direct effects on $Y$).The exposure and exposure-related phenotypes

$$X_{i}=\delta_{X}{Z_{X}}_{i}+\theta_{X}U_{i}+{\varepsilon_{X}}_{i} (3)$$

$$X_{i}^{'}=\delta_{X^{'}}{Z_{X}}_{i}+{\varepsilon_{X^{'}}}_{i} (4)$$

$$X_{i}^{*}=\kappa_{X^{*}}X_{i}+{\varepsilon_{X^{*}}}_{i} (5)$$

The outcome and outcome-related phenotypes

$$Y_{i}=\delta_{Y}{Z_{Y}}_{i}+\beta X_{i}+\theta_{Y}U_{i}+{\varepsilon_{Y}}_{i} (6)$$

$$Y_{i}^{'}=\delta_{Y^{'}}{Z_{Y}}_{i}+{\varepsilon_{Y^{'}}}_{i} (7)$$

$$Y_{i}^{*}=\lambda_{Y^{*}}Y_{i}+{\varepsilon_{Y^{*}}}_{i} (8)$$

The colliders

$$C_{i}^{'}=\delta_{X,C^{'}}{Z_{X}}_{i}+\delta_{Y,C^{'}}{Z_{Y}}_{i}+{\varepsilon_{C^{*}}}_{i} (9)$$

$$C_{i}^{*}=\kappa_{C^{*}}X_{i}+\lambda_{C^{*}}Y_{i}+{\varepsilon_{C}}_{i} (10)$$

In this model, there are $L_{X}$ SNPs with direct effects on $X$ (if $\delta_{X}\neq0$) and $X^{'}$ (if $\delta_{X^{'}}\neq0$), and $L_{Y}$ SNPs with direct effects on $Y$ (if $\delta_{Y}\neq0$) and $Y^{'}$ (if $\delta_{Y^{'}}\neq0$). Both sets of SNPs also have direct effects on $C^{'}$ (controlled by the $\delta_{X,C^{'}}$ and $\delta_{Y,C^{'}}$ parameters). $U\sim N(0,1)$ is an unmeasured confounder of the $X$-$Y$ association, with $\theta_{X}$ and $\theta_{Y}$ denoting the direct effects of $U$ on $X$ and $Y$, respectively. Moreover, $X$ and $Y$ have direct effects on $X^{*}$ and $Y^{*}$, respectively, and both have direct effects on $C^{*}$. $\beta$ is the causal effect of $X$ on $Y$ that we wish to estimate.

All SNPs are independent in the absence of assortative mating. $Z_{X}$ and $Z_{Y}$ are weighted additive allele scores, with weights $\gamma_{X}=\left\{ {\gamma_{X}}_{k} \right\}_{k=1}^{L_{X}}$ (with ${\gamma_{X}}_{k}$ being the direct effect of ${G_{X}}_{k}$ on $X$) and $\gamma_{Y}=\left\{ \gamma_{Y_{k}} \right\}_{k=1}^{L_{Y}}$ (with $\gamma_{Y_{k}}$ being the direct effect of $G_{Y_{k}}$ on $Y$) defined so that $E\left( \mathrm{var}\left( Z_{X} \right) \right)=E\left( \mathrm{var}\left( Z_{Y} \right) \right)=1$ in the absence of assortment. Similarly, the error terms $\varepsilon_{X}$, $\varepsilon_{X^{'}}$, $\varepsilon_{X^{*}}$, $\varepsilon_{Y}$, $\varepsilon_{Y^{'}}$, $\varepsilon_{Y^{*}}$, $\varepsilon_{C^{*}}$, and $\varepsilon_{C}$ were defined in such a way so that the expected value of the variance of the corresponding variables was 1. Therefore, all effect parameters can be interpreted as correlation coefficients, and consequently as the square root of the amount of variance explained ($R^{2}$). However, due to changes in genetic and phenotypic variance due to assortative mating (as illustrated in the results), the expected value of the variances get slightly greater than one (Supplementary Table 7), so this interpretation is only approximate.

The parameter $\zeta$ controls the number of generations. Setting $\zeta=1$ implies that there is one generation of parents (parental generation 1, or simply $P_{1}$) and offspring (filial population 1, or $F_{1}$). Setting $\zeta=2$ implies that the individuals in the $F_{1}$ population pair with individuals in an independent population with a similar genetic background to generate another filial population ($F_{2}$). The $F_{\zeta}$ population is divided at random into non-overlapping sets of $n_{X}$ and $n_{Y}$ individuals used to estimate SNP-$X$ and SNP-$Y$ associations, respectively.

The data-generating process can be conceptualised as follows:

1. A dataset of $n_{X}+n_{Y}$ $P_{1}$ individuals (e.g., mothers in $P_{1}$) is simulated. To simulate biallelic SNPs in Hardy-Weinberg Equilibrium, ${G_{X}}_{ik}\sim\mathrm{Binomial}(2,{\varphi_{X}}_{k})$ and ${G_{Y}}_{ij}\sim\mathrm{Binomial}(2,{\varphi_{Y}}_{j})$, with effect allele frequencies ${\varphi_{X}}_{k}\sim Uniform(0.1, 0.9)$ and ${\varphi_{Y}}_{j}\sim Uniform(0.1, 0.9)$.
2. Let $\zeta^{*}$ indicate the current generation. Therefore, at this point, $\zeta^{*}=1$.
3. The dataset corresponding to the fathers in $P_{\zeta^{*}}$ is simulated. To ensure that mothers and fathers share a similar genetic background, a $L\times L$ (where $L=L_{X}+L_{Y}$) matrix denoted by $M$ was calculated using $P_{\zeta^{*}}$ mothers’ data. Let $G=\left\{ G_{X},G_{Y} \right\}=\left\{ G_{k} \right\}_{k=1}^{L}$, so that $G_{k}=G_{X_{k}}$ if $k\leq L_{X}$, or $G_{k}=G_{Y_{L_{X}-k}}$ if $k>L_{X}$. $M_{a,b}=M_{b,a}$ denotes the Pearson correlation coefficient between $G_{a}$ and $G_{b}$. For example, if $L_{X}=L_{Y}=100$, then $M_{10,20}=\mathrm{cor}(G_{X_{10}},G_{X_{20}})$; $M_{10,110}=\mathrm{cor}\left( G_{X_{10}},G_{Y_{10}} \right)$; and $M_{110,120}=\mathrm{cor}\left( G_{Y_{10}},G_{Y_{20}} \right)$. To reduce processing time, $G_{X}$ and $G_{Y}$ of the fathers in $P_{1}$ were simulated from a multivariate normal distribution $N(0,M)$, and the resulting variables converted into genetic variants according to effect allele frequency assuming Hardy Weinberg Equilibrium, rather than simulated directly from a multivariate binomial distribution. Pilot simulations indicated that this approximation had no substantial impact on the results.
4. The phenotypes of the mothers and fathers are generated using the model outlined in equations 1-10; mothers and fathers are paired according to a given type (described below) and strength (described in the main text) of assortment.
5. The dataset corresponding to the $F_{\zeta^{*}}$ individuals is simulated. $F_{\zeta^{*}}$ SNPs were generated by combining one allele (sample at random from the allele pair) from each parent for each SNP. After generating $F_{\zeta^{*}}$ individuals’ $G_{X}$ and $G_{Y}$ in this way, the model outlined in equations 1-10 is used to generate $F_{1}$ individuals’ phenotypes.
6. If $\zeta^{*}=\zeta$, the data-generating process stops. Else, go step 7:
7. $\zeta^{*}$ is increased by 1.

1. $F_{\zeta^{*}-1}$ are used as the mothers in $P_{\zeta^{*}}$.
2. The process returns to step 3.

Our model allowed for the following types of assortative mating: a) cross-trait assortative mating on $X$ and $Y$ (Supplementary Figure 2, top-left), on $X^{'}$ and $Y^{'}$ (Supplementary Figure 2, top-centre), or on $X^{*}$ and $Y^{*}$ (Supplementary Figure 2, top-right); and b) single-trait assortative mating on $C^{'}$ (Supplementary Figure 2, middle-left), on $C^{*}$ (Supplementary Figure 2, centre), on $X$ (Supplementary Figure 2, middle-right), or on $Y$ (Supplementary Figure 2, bottom-left). Assortment was induced as described in the main text.

**1.2 Simulation scenarios**

The consequences of assortative mating on Mendelian randomization were evaluated in a simulation study. In all scenarios, $n_{X}=n_{Y}=20,000$, $\theta_{X}=\theta_{Y}=\sqrt{0.4}$ and $L_{X}=L_{Y}=H=50$, where $H$ is the number of $X$-associated SNPs used as genetic instruments. Exceptions are mentioned explicitly in tables and figures.

**1.2.1 Cross-trait assortative mating**

The first scenario considered cross-trait assortative mating on $X$ and $Y$. In this scenario, $\delta_{X}\in\{\sqrt{0.1},\sqrt{0.5} \}$, $\delta_{Y}\in\{\sqrt{0.1},\sqrt{0.5} \}$, (to evaluate the effect of heritability) $P\in\{0,0.2,0.4,0.6,0.8,1\}$ (to evaluate different levels of assortment). The remaining parameters were $\beta=\delta_{X^{'}}=\delta_{Y^{'}}=\delta_{X,C^{'}}=\delta_{Y,C^{'}}=\kappa_{X^{*}}=\kappa_{C^{*}}=\lambda_{Y^{*}}=\lambda_{C^{*}}=0$, and $\zeta=1$.

In the second scenario, there was cross-trait assortative mating on$X^{'}$ and $Y^{'}$. In this scenario, $\delta_{X^{'}}\in\{0,\sqrt{0.5} \}$, $\delta_{Y^{'}}\in\{0,\sqrt{0.5} \}$ (to evaluate the effect of heritability of those phenotypes, which influences their genetic correlation with $X$ and $Y$), $\delta_{X}=\delta_{Y}=\sqrt{0.5}$ and $P=1$. The remaining parameters were $\beta=\delta_{X,C^{'}}=\delta_{Y,C^{'}}=\kappa_{X^{*}}=\kappa_{C^{*}}=\lambda_{Y^{*}}=\lambda_{C^{*}}=0$, and $\zeta=1$.

In the third scenario, there was cross-trait assortative mating on$X^{*}$ and $Y^{*}$. In this scenario, $\kappa_{X^{*}}\in\{0,\sqrt{0.5} \}$, $\lambda_{Y^{*}}\in\{0,\sqrt{0.5} \}$ (to evaluate the effect of different direct effects of $X$ and $Y$ on those traits), $\delta_{X}=\delta_{Y}=\sqrt{0.5}$ and $P=1$. The remaining parameters were$\beta=\delta_{X^{'}}=\delta_{Y^{'}}=\delta_{X,C^{'}}=\delta_{Y,C^{'}}=\kappa_{C^{*}}=\lambda_{C^{*}}=0$, and $\zeta=1$.

**1.2.2 Single-trait assortative mating**

The fourth scenario considered single-trait assortative mating on $C^{'}$. In this scenario, $\delta_{X,C^{'}}\in\{0,\sqrt{0.4} \}$, $\delta_{Y,C^{'}}\in\{0,\sqrt{0.4} \}$ (to evaluate the effect of the genetic correlation of $C^{'}$ with $X$ and $Y$), $\delta_{X}=\delta_{Y}=\sqrt{0.5}$ and $P=1$. The remaining parameters were $\beta=\delta_{X^{'}}=\delta_{Y^{'}}=\kappa_{X^{*}}=\kappa_{C^{*}}=\lambda_{Y^{*}}=\lambda_{C^{*}}=0$, and $\zeta=1$.

In the fifth scenario, there was single-trait assortative mating on $C^{*}$. In this scenario, $\kappa_{C^{*}}\in\{0,\sqrt{0.3} \}$, $\lambda_{C^{*}}\in\{0,\sqrt{0.3} \}$ (to evaluate the effect of different direct effects of $X$ and $Y$ on $C^{*}$), $\delta_{X}=\delta_{Y}=\sqrt{0.5}$ and $P=1$. The remaining parameters were $\beta=\delta_{X^{'}}=\delta_{Y^{'}}=\delta_{X,C^{'}}=\delta_{Y,C^{'}}=\kappa_{X^{*}}=\lambda_{Y^{*}}=0$, and $\zeta=1$.

In scenarios six and seven, there was single-trait assortative mating on $X$ or on $Y$. In these scenarios, $\delta_{X}=\delta_{Y}=\sqrt{0.5}$ and $P=1$. The remaining parameters were $\beta=\delta_{X^{'}}=\delta_{Y^{'}}=\delta_{X,C^{'}}=\delta_{Y,C^{'}}=\kappa_{X^{*}}=\kappa_{C^{*}}=\lambda_{Y^{*}}=\lambda_{C^{*}}=0$, and $\zeta=1$.

**1.2.3 Cross-trait assortative mating along generations**

Scenario 8 evaluates the cumulative effect of cross-trait assortative on $X$ and $Y$ mating along generations. To do so, $\zeta\in\{1,2,\ldots,10\}$. Moreover, $P\in\{0.5,1\}$, $\delta_{X}\in\left\{ \sqrt{0.1}, \sqrt{0.5} \right\}$ and $\delta_{Y}\in\left\{ \sqrt{0.1}, \sqrt{0.5} \right\}$. The remaining parameters were $\beta=\delta_{X^{'}}=\delta_{Y^{'}}=\delta_{X,C^{'}}=\delta_{Y,C^{'}}=\kappa_{X^{*}}=\kappa_{C^{*}}=\lambda_{Y^{*}}=\lambda_{C^{*}}=0$.

**1.2.4 Power to detect a causal effect**

Scenario 9 evaluates the power of selected methods to detect a causal effect $\beta=0.05$ in the absence of assortative mating ($P=0$). $\delta_{X}\in\left\{ \sqrt{0.1}, \sqrt{0.2} \right\}$ and $\delta_{Y}\in\left\{ \sqrt{0.1}, \sqrt{0.2} \right\}$. The remaining parameters were $\delta_{X^{'}}=\delta_{Y^{'}}=\delta_{X,C^{'}}=\delta_{Y,C^{'}}=\kappa_{X^{*}}=\kappa_{C^{*}}=\lambda_{Y^{*}}=\lambda_{C^{*}}=0$, and $\zeta=1$.

**1.3 Statistical analyses**

In addition to the four versions of the two-stage least squares regression method described in the main text, we also investigated the bias and the coverage of four summary-data MR estimators (described below). In all of them, inverse-variance weights were derived under the No Measurement Error (NOME) assumption. Those methods require regression coefficients and standard errors of the association of $X$ with each genetic instrument, and of $Y$ with each genetic instrument. Those were estimated by regressing $X$ on each genetic variant in $G_{X}$ in one random half of the data, and by regressing$Y$ on each genetic variant in $G_{X}$ in the other half.

- Inverse-variance weighting (IVW): this corresponds to a weighted average of the all individual-instrument ratio estimates (ie, the quotient of the instrument-outcome effect estimate divided by the instrument-exposure effect estimate). The method assumes that all genetic instruments are valid, or that horizontal pleiotropy balances out. The multiplicative random effects version of the IVW method was used (Bowden et al., 2017a; Burgess, Butterworth, & Thompson, 2013).
- MR-Egger regression: this method is implemented as a weighted linear regression of instrument-outcome on instrument-exposure effect estimates. The method is consistent even if all instruments are invalid, as long as the Instrument Strength Independent of Direct Effects (InSIDE) assumption holds and the exposure has the same effect on the outcome in all subgroups of the population. Moreover, the intercept of this regression can be used as a test of overall directional horizontal pleiotropy. The multiplicative random effects version of the method was used (Bowden, Davey Smith, & Burgess, 2015; Bowden et al., 2017a).
- Weighted median: this method uses the median of the weighted empirical distribution function as the causal effect estimate. It assumes that up (but not including) 50% of the weights come from invalid instruments (Bowden, Davey Smith, Haycock, & Burgess, 2016).
- Mode-based estimate (MBE): this method uses the mode of the weighted empirical density function (computed using a normal kernel) as the causal effect estimate. Consistent estimation using this method requires the ZEro Modal Pleitropy Assumption (ZEMPA), which means that the largest (with regards to the weights) subset of instruments that estimate the same causal effect parameter is the subset of valid instruments (Hartwig, Davey Smith, & Bowden, 2017).

MR-Egger intercept estimate and power, and the $\chi^{2}$ statistic and power of the Cochran’s Q test of heterogeneity (Greco, Minelli, Sheehan, & Thompson, 2015) using modified 2^nd^ order weights (which improve the false-rejection rate of the test) (Bowden et al., 2017b) (both commonly used to detect horizontal pleiotropy) were also calculated.

**SUPPLEMENTARY REFERENCES**

Bowden J, Davey Smith G, Burgess S. (2015). Mendelian randomization with invalid instruments: effect estimation and bias detection through Egger regression. *Int J Epidemiol*, 44, 512-25.

Bowden J, Davey Smith G, Haycock PC, Burgess S. (2016). Consistent Estimation in Mendelian Randomization with Some Invalid Instruments Using a Weighted Median Estimator. *Genet Epidemiol*, 40, 304-14.

Bowden J, Del Greco M F, Minelli C, Davey Smith G, Sheehan NA, Thompson JR. (2017a). A framework for the investigation of pleiotropy in two-sample summary data Mendelian randomization. *Stat Med*, 36, 1783-802.

Bowden J, Greco MF, Minelli C, Lawlor DA, Sheehan N, Thompson J, Davey Smith G. (2017b). Improving the accuracy of two-sample summary data Mendelian randomization: moving beyond the NOME assumption. *bioRxiv*, doi: 10.1101/159442.

Burgess S, Butterworth A, Thompson SG. (2013). Mendelian randomization analysis with multiple genetic variants using summarized data. *Genet Epidemiol*, 37, 658-65.

Greco MF, Minelli C, Sheehan NA, Thompson JR. (2015). Detecting pleiotropy in Mendelian randomisation studies with summary data and a continuous outcome. *Stat Med*, 34, 2926-40.

Hartwig FP, Davey Smith G, Bowden J. (2017). Robust inference in summary data Mendelian randomization via the zero modal pleiotropy assumption. *Int J Epidemiol*, 46, 1985-98.

**SUPPLEMENTARY TABLES**

**Supplementary Table 1.** Bias and false-rejection rates (FRR) of two-stage least squares (TSLS) regression methods in the presence of cross-trait assortative mating on $X$ and $Y$ over generations (scenario 8) under the causal null (i.e., $\beta=0$).

| $\boldsymbol{P}$ | **Number of** | **Bias** | | | **FRR (%)** | | |
| --- | --- | --- | --- | --- | --- | --- | --- |
|  | **generations** | **TSLS (1)** | **TSLS (2)** | **TSLS (3)** | **TSLS (1)** | **TSLS (2)** | **TSLS (3)** |
| 0.5 | 1 | 0.032 | 0.001 | 0.001 | 88.9 | 5.3 | 5.4 |
|  | 3 | 0.051 | 0.001 | 0.001 | 99.9 | 5.2 | 5.1 |
|  | 5 | 0.055 | 0.001 | 0.001 | 100.0 | 5.1 | 5.3 |
|  | 7 | 0.056 | 0.001 | 0.001 | 99.9 | 5.0 | 5.1 |
|  | 9 | 0.056 | 0.001 | 0.001 | 100.0 | 4.5 | 4.6 |
| 1 | 1 | 0.126 | 0.001 | 0.001 | 100.0 | 5.3 | 5.3 |
|  | 3 | 0.200 | 0.001 | 0.001 | 100.0 | 5.1 | 4.9 |
|  | 5 | 0.222 | 0.001 | 0.001 | 100.0 | 4.8 | 4.7 |
|  | 7 | 0.229 | 0.001 | 0.001 | 100.0 | 5.0 | 4.8 |
|  | 9 | 0.232 | 0.001 | 0.001 | 100.0 | 5.4 | 5.1 |

TSLS (1): no covariates. TSLS (2): adjusting for parental allele scores. TSLS (3): adjusting for parental non-transmitted allele scores.

In all cases, $h_{X}^{2}=h_{Y}^{2}=50\%$.

$P$: degree of assortment.

**Supplementary Table 2.** Bias and false-rejection rates (FRR) of Mendelian randomization methods in the presence of cross-trait assortative mating on $X^{'}$ and $Y^{'}$ (scenario 2), or on $X^{*}$ and $Y^{*}$ (scenario 3) under the causal null (i.e., $\beta=0$).

| **Parameters** | **Statistic** | **Method** | | | | | | |
| --- | --- | --- | --- | --- | --- | --- | --- | --- |
|  |  | **TSLS (1)** | **TSLS (2)** | **TSLS (3)** | **IVW** | **MR-Egger** | **Weighted Median** | **MBE** |
| **Scenario 2 (**$\boldsymbol{Z}_{\boldsymbol{X}}\boldsymbol{X}^{\boldsymbol{'}}$**: direct effect of** $\boldsymbol{Z}_{\boldsymbol{X}}$ **on** $\boldsymbol{X}^{\boldsymbol{'}}$**;** $\boldsymbol{Z}_{\boldsymbol{Y}}\boldsymbol{Y}^{\boldsymbol{'}}$**: direct effect of** $\boldsymbol{Z}_{\boldsymbol{Y}}$ **on** $\boldsymbol{Y}^{\boldsymbol{'}}$**)** | | | | | | | | |
| $Z_{X}X^{'}$: absent | Bias | 0.001 | 0.001 | 0.001 | 0.000 | 0.000 | 0.000 | 0.000 |
| $Z_{Y}Y^{'}$: absent | FRR (%) | 5.4 | 5.1 | 5.2 | 3.7 | 4.4 | 2.4 | 0.5 |
| $Z_{X}X^{'}$: absent | Bias | 0.001 | 0.001 | 0.001 | 0.000 | 0.000 | 0.000 | 0.000 |
| $Z_{Y}Y^{'}$: present | FRR (%) | 5.2 | 5.0 | 5.0 | 3.7 | 3.6 | 2.4 | 0.4 |
| $Z_{X}X^{'}$: present | Bias | 0.001 | 0.001 | 0.001 | 0.000 | 0.000 | 0.000 | -0.001 |
| $Z_{Y}Y^{'}$: absent | FRR (%) | 5.8 | 5.3 | 5.3 | 4.1 | 3.6 | 2.7 | 0.5 |
| $Z_{X}X^{'}$: present | Bias | 0.126 | 0.001 | 0.001 | 0.125 | 0.121 | 0.124 | 0.124 |
| $Z_{Y}Y^{'}$: present | FRR (%) | 100.0 | 5.2 | 4.9 | 100.0 | 94.0 | 100.0 | 99.8 |
| **Scenario 3 (**$\boldsymbol{X}\boldsymbol{X}^{\boldsymbol{*}}$**: direct effect of** $\boldsymbol{X}$ **on** $\boldsymbol{X}^{\boldsymbol{*}}$**;** $\boldsymbol{Y}\boldsymbol{Y}^{\boldsymbol{*}}$**: direct effect of** $\boldsymbol{Y}$ **on** $\boldsymbol{Y}^{\boldsymbol{*}}$**)** | | | | | | | | |
| $XX^{*}$: absent | Bias | 0.001 | 0.001 | 0.001 | 0.000 | 0.001 | 0.000 | 0.000 |
| $YY^{*}$: absent | FRR (%) | 5.4 | 4.9 | 4.9 | 3.7 | 4.4 | 2.2 | 0.2 |
| $XX^{*}$: absent | Bias | 0.001 | 0.001 | 0.001 | 0.000 | 0.001 | 0.000 | 0.000 |
| $YY^{*}$: present | FRR (%) | 5.8 | 5.5 | 5.5 | 3.9 | 3.9 | 2.6 | 0.4 |
| $XX^{*}$: present | Bias | 0.001 | 0.001 | 0.001 | 0.000 | 0.001 | 0.000 | 0.000 |
| $YY^{*}$: absent | FRR (%) | 5.6 | 5.3 | 5.3 | 4.4 | 4.5 | 2.5 | 0.4 |
| $XX^{*}$: present | Bias | 0.063 | 0.001 | 0.001 | 0.062 | 0.060 | 0.062 | 0.061 |
| $YY^{*}$: present | FRR (%) | 100.0 | 5.1 | 5.3 | 100.0 | 40.2 | 99.3 | 64.1 |

IVW: inverse-variance weighting. MBE: mode-based estimate.

$Z_{X}X^{'}$ and $Z_{Y}Y^{'}$ correspond to parameters $\delta_{X^{'}}$ and $\delta_{Y^{'}}$, respectively. $XX^{*}$ and $YY^{*}$ correspond to parameters $\kappa_{X^{*}}$ and $\lambda_{Y^{*}}$, respectively (see the description of the simulation model in the Supplementary Methods for details).

**Supplementary Table 3.** Bias and false-rejection rates (FRR) of Mendelian randomization methods in the presence of single-trait assortative mating on $C^{'}$ (scenario 4) or $C^{*}$ (scenario 5) under the causal null (i.e., $\beta=0$).

| **Parameters** | **Statistic** | **Method** | | | | | | |
| --- | --- | --- | --- | --- | --- | --- | --- | --- |
|  |  | **TSLS (1)** | **TSLS (2)** | **TSLS (3)** | **IVW** | **MR-Egger** | **Weighted Median** | **MBE** |
| **Scenario 4 (**$\boldsymbol{Z}_{\boldsymbol{X}}\boldsymbol{C}^{\boldsymbol{'}}$**: direct effect of** $\boldsymbol{Z}_{\boldsymbol{X}}$ **on** $\boldsymbol{C}^{\boldsymbol{'}}$**;** $\boldsymbol{Z}_{\boldsymbol{Y}}\boldsymbol{C}^{\boldsymbol{'}}$**: direct effect of** $\boldsymbol{Z}_{\boldsymbol{Y}}$ **on** $\boldsymbol{C}^{\boldsymbol{'}}$**)** | | | | | | | | |
| $Z_{X}C^{'}$: absent | Bias | 0.001 | 0.001 | 0.001 | 0.000 | -0.001 | 0.000 | 0.000 |
| $Z_{Y}C^{'}$: absent | FRR (%) | 6.0 | 5.2 | 5.2 | 3.9 | 3.7 | 2.9 | 0.6 |
| $Z_{X}C^{'}$: absent | Bias | 0.001 | 0.001 | 0.001 | 0.000 | 0.000 | 0.000 | 0.000 |
| $Z_{Y}C^{'}$: present^a^ | FRR (%) | 5.6 | 5.4 | 5.4 | 3.9 | 3.9 | 3.4 | 0.4 |
| $Z_{X}C^{'}$: present^a^ | Bias | 0.001 | 0.001 | 0.001 | 0.000 | 0.000 | 0.000 | 0.000 |
| $Z_{Y}C^{'}$: absent | FRR (%) | 5.3 | 4.9 | 4.9 | 6.2 | 3.8 | 3.9 | 0.4 |
| $Z_{X}C^{'}$: present^a^ | Bias | 0.167 | 0.000 | 0.001 | 0.167 | 0.162 | 0.166 | 0.166 |
| $Z_{Y}C^{'}$: present^a^ | FRR (%) | 100.0 | 5.6 | 5.3 | 100.0 | 99.9 | 100.0 | 100.0 |
| **Scenario 5 (**$\boldsymbol{X}\boldsymbol{C}^{\boldsymbol{*}}$**: direct effect of** $\boldsymbol{X}$ **on** $\boldsymbol{C}^{\boldsymbol{*}}$**;** $\boldsymbol{Y}\boldsymbol{C}^{\boldsymbol{*}}$**: direct effect of** $\boldsymbol{Y}$ **on** $\boldsymbol{C}^{\boldsymbol{*}}$**)** | | | | | | | | |
| $XC^{*}$: absent | Bias | 0.001 | 0.001 | 0.001 | 0.000 | 0.000 | 0.000 | 0.000 |
| $YC^{*}$: absent | FRR (%) | 5.3 | 5.1 | 5.1 | 4.2 | 4.1 | 2.8 | 0.5 |
| $XC^{*}$: absent | Bias | 0.001 | 0.001 | 0.001 | 0.000 | 0.000 | 0.000 | 0.000 |
| $YC^{*}$: present^b^ | FRR (%) | 5.6 | 5.0 | 5.0 | 4.2 | 4.3 | 2.4 | 0.4 |
| $XC^{*}$: present^b^ | Bias | 0.001 | 0.001 | 0.001 | 0.000 | 0.000 | 0.000 | 0.000 |
| $YC^{*}$: absent | FRR (%) | 5.4 | 4.7 | 4.7 | 4.9 | 3.9 | 3.2 | 0.4 |
| $XC^{*}$: present^b^ | Bias | 0.070 | 0.001 | 0.001 | 0.069 | 0.067 | 0.069 | 0.069 |
| $YC^{*}$: present^b^ | FRR (%) | 100.0 | 4.8 | 4.9 | 100.0 | 53.3 | 100.0 | 80.6 |

IVW: inverse-variance weighting. MBE: mode-based estimate.

$Z_{X}C^{'}$ and $Z_{Y}C^{'}$ correspond to the $\delta_{X,C^{'}}$ and $\delta_{Y,C^{'}}$, respectively. $XC^{*}$ and $YC^{*}$ correspond to the $\kappa_{C^{*}}$ and $\lambda_{C^{*}}$, respectively (see the description of the simulation model in the Supplementary Materials for details).

^a^$\sqrt{0.4}$. ^b^$\sqrt{0.3}$.

**Supplementary Table 4.** Bias and false-rejection rates (FRR) of Mendelian randomization methods in the presence of single-trait assortative mating on $X$ (scenario 6), or $Y$ (scenario 7) under the causal null (i.e., $\beta=0$).

| **Scenario** | **Method** | **Statistic** | |
| --- | --- | --- | --- |
|  |  | **Bias** | **FRR (%)** |
| 6 | TSLS (1) | 0.001 | 5.4 |
|  | TSLS (2) | 0.001 | 5.0 |
|  | TSLS (3) | 0.001 | 5.2 |
|  | IVW | 0.000 | 7.2 |
|  | MR-Egger | 0.000 | 3.9 |
|  | Weighted median | 0.000 | 3.6 |
|  | MBE | 0.000 | 0.5 |
| 7 | TSLS (1) | 0.001 | 6.1 |
|  | TSLS (2) | 0.001 | 5.4 |
|  | TSLS (3) | 0.001 | 5.3 |
|  | IVW | 0.000 | 4.0 |
|  | MR-Egger | 0.000 | 4.3 |
|  | Weighted median | 0.000 | 2.4 |
|  | MBE | 0.000 | 0.5 |

TSLS: two-stage least squares regression. TSLS (1): no covariates. TSLS (2): adjusting for parental allele scores. TSLS (3): adjusting for parental non-transmitted allele scores. IVW: inverse-variance weighting. MBE: mode-based estimate.

**Supplementary Table 5.** Performance of different tests to detect assortative mating bias.

| **Scenario^a^** | **Statistic** | **MR-Egger** | **Cochran’s** | **TSLS (2)** | **TSLS (2)** | **TSLS (3)** | **TSLS (3)** |
| --- | --- | --- | --- | --- | --- | --- | --- |
|  |  | **intercept** | **Q** | **parent 1** | **parent 2** | **parent 1** | **parent 2** |
| Scenario 1 | Estimate | 0.001 | 50.596 | 0.124 | 0.124 | 0.104 | 0.104 |
| (assortment on $X$ and $Y$) | Power (%) | 4.2 | 7.4 | 100.0 | 100.0 | 100.0 | 100.0 |
| Scenario 2 | Estimate | 0.001 | 50.419 | 0.124 | 0.124 | 0.125 | 0.125 |
| (assortment on $X^{'}$ and $Y^{'}$) | Power (%) | 4.4 | 6.1 | 100.0 | 100.0 | 100.0 | 100.0 |
| Scenario 3 | Estimate | 0.000 | 50.382 | 0.062 | 0.062 | 0.057 | 0.057 |
| (assortment on $X^{*}$ and $Y^{*}$) | Power (%) | 4.2 | 6.9 | 99.9 | 99.9 | 97.1 | 97.2 |
| Scenario 4 | Estimate | 0.001 | 51.078 | 0.120 | 0.120 | 0.143 | 0.143 |
| (assortment on $C^{'}$) | Power (%) | 4.5 | 7.6 | 100.0 | 100.0 | 100.0 | 100.0 |
| Scenario 5 | Estimate | 0.000 | 50.460 | 0.060 | 0.061 | 0.053 | 0.053 |
| (assortment on $C^{*}$) | Power (%) | 4.5 | 7.1 | 100.0 | 99.9 | 80.9 | 80.1 |
| Scenario 6 | Estimate | 0.000 | 50.198 | 0.000 | 0.000 | 0.000 | -^c^ |
| (assortment on $X$) | Power (%)^b^ | 3.2 | 6.6 | 5.1 | 5.4 | 5.4 | -^c^ |
| Scenario 7 | Estimate | 0.000 | 52.048 | 0.000 | 0.000 | 0.000 | 0.000 |
| (assortment on $Y$) | Power (%)^b^ | 3.8 | 9.7 | 5.0 | 5.0 | 4.9 | 5.3 |
| Scenario 8 (10 generations) | Estimate | 0.003 | 51.145 | 0.208 | 0.180 | 0.194 | 0.156 |
| (assortment on $X$ and $Y$) | Power (%) | 6.5 | 7.9 | 100.0 | 100.0 | 100.0 | 100.0 |
| Scenario 9 | Estimate | 0.001 | 49.551 | 0.001 | 0.000 | 0.000 | 0.000 |
| (no assortment) | Power (%)^b^ | 4.2 | 5.5 | 5.0 | 5.1 | 4.9 | 4.9 |

^a^In all cases, $P$=1 (ie, maximum degree of assorment). See the description of the simulation model in the Supplementary Materials for further details.

^b^In those scenarios there is no assortative mating bias, so power can be interpreted as the false-rejection rate.

**Supplementary Table 6.** Summary of phenotypic and genotypic variables related to height and education in ALSPAC mother-father-offspring trios.

|  | **Trio member** | **Trait** | **N** | **Mean** | **SD** | **Min** | **Max** |
| --- | --- | --- | --- | --- | --- | --- | --- |
| Phenotypes | Offspring | Height | 1,170 | 125.77 | 5.54 | 108.10 | 147.30 |
|  |  | Education | 1,170 | 44.41 | 7.69 | 10.00 | 61.21 |
|  | Mother | Height | 1,113 | 164.58 | 6.66 | 147.32 | 185.42 |
|  |  | Education | 1,127 | 13.53 | 4.09 | 7.00 | 20.00 |
|  | Father | Height | 989 | 1.80 | 0.07 | 1.57 | 2.13 |
|  |  | Education | 1,125 | 14.78 | 4.64 | 7.00 | 20.00 |
| Allele scores | Offspring | Height | 1,170 | -0.02 | 0.48 | -1.74 | 1.50 |
|  |  | Education | 1,170 | 0.20 | 0.16 | -0.35 | 0.79 |
|  | Mother | Height | 1,170 | -0.03 | 0.49 | -1.82 | 1.64 |
|  |  | Education | 1,170 | 0.19 | 0.16 | -0.32 | 0.64 |
|  | Father | Height | 1,170 | 0.00 | 0.47 | -1.57 | 1.48 |
|  |  | Education | 1,170 | 0.13 | 0.15 | -0.37 | 0.66 |

ALSPAC: Avon Longitudinal Study of Parents and Children. N: sample size. SD: standard deviation.

**Supplementary Table 7.** Pearson correlation between mothers’ exposure $X$ and fathers’ outcome $Y$ in the presence of cross-trait assortative mating on $X$ and $Y$ (scenarios 1 and 8).

| **Scenario** | **Number of generations** | $\boldsymbol{P}$ | $\boldsymbol{h}_{\boldsymbol{X}}^{\boldsymbol{2}}$ | $\boldsymbol{h}_{\boldsymbol{Y}}^{\boldsymbol{2}}$ | $\boldsymbol{r(}\mathbf{mothers'}\boldsymbol{X,}$  $\mathbf{father}\mathbf{s}^{\mathbf{'}}\boldsymbol{Y)}$ |
| --- | --- | --- | --- | --- | --- |
| 1 | 1 | 0 | 10% | 10% | 0.00 |
|  |  | 0 |  | 50% | 0.00 |
|  |  | 0 | 50% | 10% | 0.00 |
|  |  | 0 |  | 50% | 0.00 |
|  |  | 0.2 | 10% | 10% | 0.02 |
|  |  | 0.2 |  | 50% | 0.02 |
|  |  | 0.2 | 50% | 10% | 0.02 |
|  |  | 0.2 |  | 50% | 0.02 |
|  |  | 0.4 | 10% | 10% | 0.09 |
|  |  | 0.4 |  | 50% | 0.09 |
|  |  | 0.4 | 50% | 10% | 0.09 |
|  |  | 0.4 |  | 50% | 0.09 |
|  |  | 0.6 | 10% | 10% | 0.21 |
|  |  | 0.6 |  | 50% | 0.21 |
|  |  | 0.6 | 50% | 10% | 0.21 |
|  |  | 0.6 |  | 50% | 0.21 |
|  |  | 0.8 | 10% | 10% | 0.37 |
|  |  | 0.8 |  | 50% | 0.37 |
|  |  | 0.8 | 50% | 10% | 0.37 |
|  |  | 0.8 |  | 50% | 0.37 |
|  |  | 1 | 10% | 10% | 0.58 |
|  |  | 1 |  | 50% | 0.58 |
|  |  | 1 | 50% | 10% | 0.58 |
|  |  | 1 |  | 50% | 0.58 |
| 8 | 1 | 0.5 | 10% | 10% | 0.15 |
|  |  |  |  | 50% | 0.14 |
|  |  |  | 50% | 10% | 0.14 |
|  |  |  |  | 50% | 0.14 |
|  | 3 |  | 10% | 10% | 0.14 |
|  |  |  |  | 50% | 0.15 |
|  |  |  | 50% | 10% | 0.15 |
|  |  |  |  | 50% | 0.15 |
|  | 5 |  | 10% | 10% | 0.15 |
|  |  |  |  | 50% | 0.15 |
|  |  |  | 50% | 10% | 0.15 |
|  |  |  |  | 50% | 0.15 |
|  | 7 |  | 10% | 10% | 0.15 |
|  |  |  |  | 50% | 0.15 |
|  |  |  | 50% | 10% | 0.15 |
|  |  |  |  | 50% | 0.15 |
|  | 9 |  | 10% | 10% | 0.15 |
|  |  |  |  | 50% | 0.15 |
|  |  |  | 50% | 10% | 0.15 |
|  |  |  |  | 50% | 0.15 |
|  | 1 | 1 | 10% | 10% | 0.58 |
|  |  |  |  | 50% | 0.58 |
|  |  |  | 50% | 10% | 0.58 |
|  |  |  |  | 50% | 0.58 |
|  | 3 |  | 10% | 10% | 0.58 |
|  |  |  |  | 50% | 0.59 |
|  |  |  | 50% | 10% | 0.59 |
|  |  |  |  | 50% | 0.61 |
|  | 5 |  | 10% | 10% | 0.58 |
|  |  |  |  | 50% | 0.59 |
|  |  |  | 50% | 10% | 0.59 |
|  |  |  |  | 50% | 0.62 |
|  | 7 |  | 10% | 10% | 0.58 |
|  |  |  |  | 50% | 0.59 |
|  |  |  | 50% | 10% | 0.59 |
|  |  |  |  | 50% | 0.62 |
|  | 9 |  | 10% | 10% | 0.58 |
|  |  |  |  | 50% | 0.59 |
|  |  |  | 50% | 10% | 0.59 |
|  |  |  |  | 50% | 0.62 |

$h_{X}^{2}$: narrow-sense heritability of $X$.

$h_{Y}^{2}$: narrow-sense heritability of $Y$.

**Supplementary Table 8.** Genetic^a^ and phenotypic variances according to cross-trait assortative mating on $X$ and $Y$ over generations (scenario 8).

| $\boldsymbol{P}$ | **Number of generations** | $\boldsymbol{Z}_{\boldsymbol{X}}$ | $\boldsymbol{Z}_{\boldsymbol{Y}}$ | $\boldsymbol{X}$ | $\boldsymbol{Y}$ |
| --- | --- | --- | --- | --- | --- |
| 0.5 | 1 | 1.001 | 0.999 | 1.002 | 0.998 |
|  | 3 | 1.002 | 1.002 | 1.004 | 1.005 |
|  | 5 | 1.002 | 1.002 | 1.004 | 1.005 |
|  | 7 | 1.003 | 1.003 | 1.005 | 1.006 |
|  | 9 | 1.002 | 1.002 | 1.005 | 1.004 |
| 1 | 1 | 0.999 | 1.000 | 0.998 | 1.001 |
|  | 3 | 1.026 | 1.024 | 1.051 | 1.049 |
|  | 5 | 1.039 | 1.041 | 1.078 | 1.081 |
|  | 7 | 1.046 | 1.045 | 1.091 | 1.090 |
|  | 9 | 1.047 | 1.048 | 1.095 | 1.096 |

^a^Here, $Z_{X}$ and $Z_{Y}$ denote unweighted allele scores.

In all cases, In all cases, $h_{X}^{2}=h_{Y}^{2}=50\%$.

**Supplementary Table 9.** Pearson correlation coefficients ($r$) between genetic variables according to cross-trait assortative mating on $X$ and $Y$ over generations (scenario 8).

| $\boldsymbol{P}$ | **Number of generations** | $\boldsymbol{r(}\boldsymbol{Z}_{\boldsymbol{X}}\boldsymbol{,}\boldsymbol{Z}_{\boldsymbol{Y}}\boldsymbol{)}$**^a^** | $\mathbf{mean}\left[ \boldsymbol{r}\left( {\boldsymbol{G}_{\boldsymbol{X}}}_{\boldsymbol{k}^{\boldsymbol{'}}}\boldsymbol{,}{\boldsymbol{G}_{\boldsymbol{X}}}_{\boldsymbol{k}^{\boldsymbol{*}}} \right) \right]$**^b^** | $\mathbf{mean}\left[ \boldsymbol{r}\left( {\boldsymbol{G}_{\boldsymbol{Y}}}_{\boldsymbol{k}^{\boldsymbol{'}}}\boldsymbol{,}{\boldsymbol{G}_{\boldsymbol{Y}}}_{\boldsymbol{k}^{\boldsymbol{*}}} \right) \right]$**^c^** |
| --- | --- | --- | --- | --- |
| 0.5 | 1 | 0.028 | 0.0000 | 0.0000 |
|  | 3 | 0.046 | 0.0001 | 0.0001 |
|  | 5 | 0.049 | 0.0001 | 0.0001 |
|  | 7 | 0.050 | 0.0001 | 0.0001 |
|  | 9 | 0.050 | 0.0001 | 0.0001 |
| 1 | 1 | 0.113 | 0.0000 | 0.0000 |
|  | 3 | 0.181 | 0.0009 | 0.0009 |
|  | 5 | 0.201 | 0.0014 | 0.0014 |
|  | 7 | 0.209 | 0.0017 | 0.0017 |
|  | 9 | 0.212 | 0.0018 | 0.0018 |

^a^Here, $Z_{X}$ and $Z_{Y}$ denote unweighted allele scores.

^b^This can be interpreted as the average correlation between all pairs of genetic variants with direct effects on $X$.

^c^This can be interpreted as the average correlation between all pairs of genetic variants with direct effects on $Y$.

In all cases, In all cases, $h_{X}^{2}=h_{Y}^{2}=50\%$.

$P$: degree of assortment.

**SUPPLEMENTARY FIGURE LEGENDS**

**Supplementary Figure 1.** **Causal diagrams depicting a causal structure corresponding to mother-father-offspring trios and cross-trait assortative mating on** $\boldsymbol{X}$ **and** $\boldsymbol{Y}$**.**

$X:$ exposure phenotype. $Y$: outcome phenotype. $U$: unmeasured common cause of $X$ and $Y$. $G_{X}^{M}$ and $G_{X}^{U}$: collection of measured and unmeasured (respectively) genetic variants with direct effects on $X$. $G_{Y}^{M}$ and $G_{Y}^{U}$: collection of measured and unmeasured (respectively) genetic variants with direct effects on $Y$.

**Supplementary Figure 2. Directed acyclic graphs (DAGs) depicting the causal structures corresponding to each simulation scenario.**

Phenotypes under assortment are circled: two circled variables mean that they are under cross-trait assortative mating. $Z_{X}$ and $Z_{Y}$: allele score composed of $X$-associated and $Y$-associated genetic variants, respectively. $X$ and $Y$: exposure and outcome phenotypes, respectively. U: unmeasured common cause of $X$ and $Y$. $X^{'}$ and $X^{*}$: unmeasured phenotypes associated with $X$ through horizontal and vertical pleiotropy, respectively. $Y^{'}$ and $Y^{*}$: same as $X^{'}$ and $X^{*}$, but with respect to $Y$. $C^{'}$: collider of $Z_{X}$ and $Z_{Y}$. $C^{*}$: collider of $X$ and $Y$.

**Supplementary Figure 3. Bias and false-rejection rates of summary data Mendelian randomization methods in the presence of cross-trait assortative mating on** $\boldsymbol{X}$ **and** $\boldsymbol{Y}$ **(scenario 1) under the causal null (i.e.,** $\boldsymbol{\beta=0}$**), for different levels of assortment (**$\boldsymbol{P}$**) and narrow-sense heritability of** $\boldsymbol{X}$ **(**$\boldsymbol{h}_{\boldsymbol{X}}^{\boldsymbol{2}}$**) and** $\boldsymbol{Y}$ **(**$\boldsymbol{h}_{\boldsymbol{Y}}^{\boldsymbol{2}}$**).**

IVW: inverse-variance weighting. W. Median: weighted median. MBE: mode-based estimate.
