# Supplementary figures and images for: Bias in Mendelian randomization due to assortative mating
Source: Genet Epidemiol. 2018 Jul 3;42(7):608–20. doi: 10.1002/gepi.22138 (PMC6221130; doi:10.1002/gepi.22138)

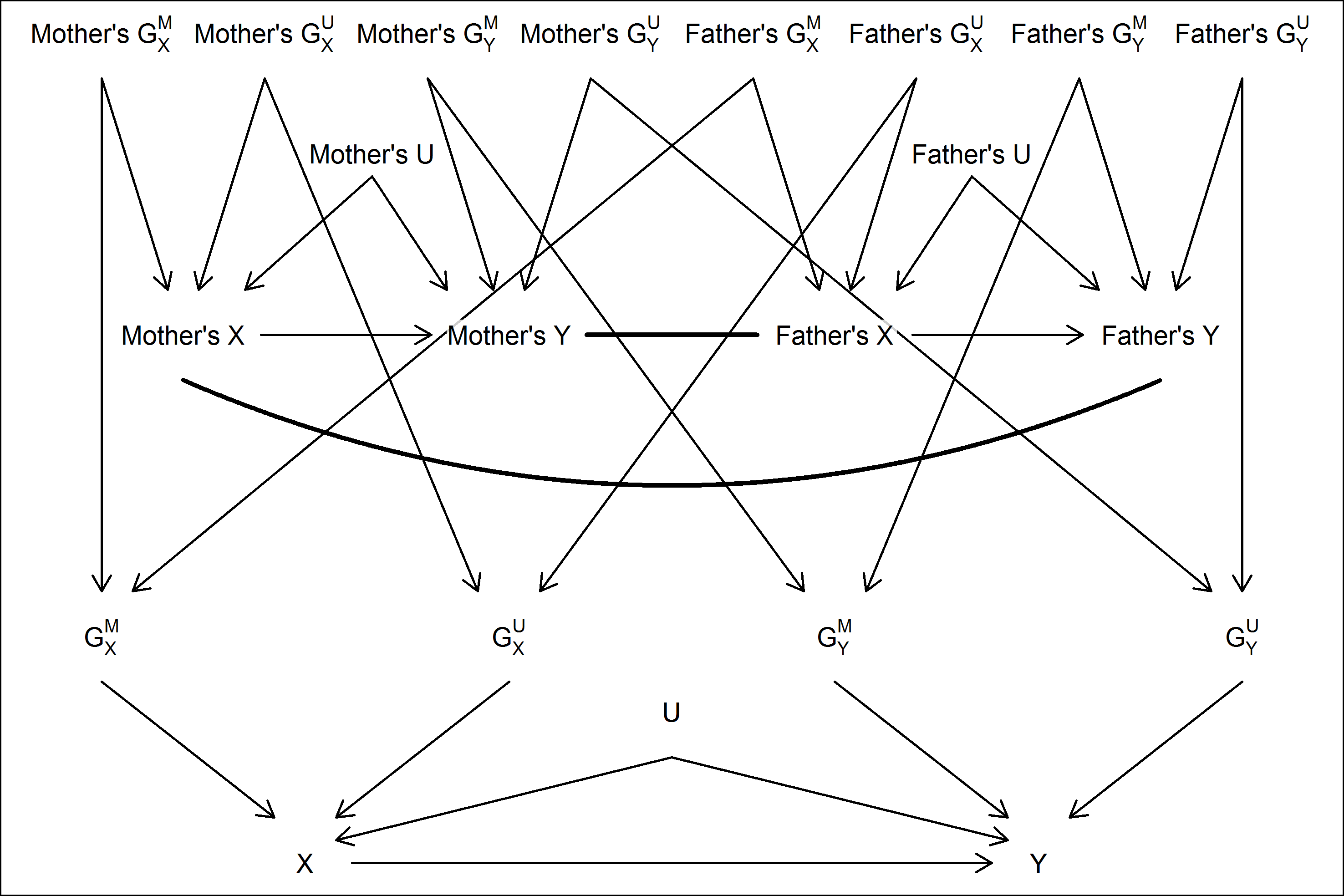

Supplement: Supplementary file 2 — Supporting Information [file GEPI-42-608-s002.tiff]

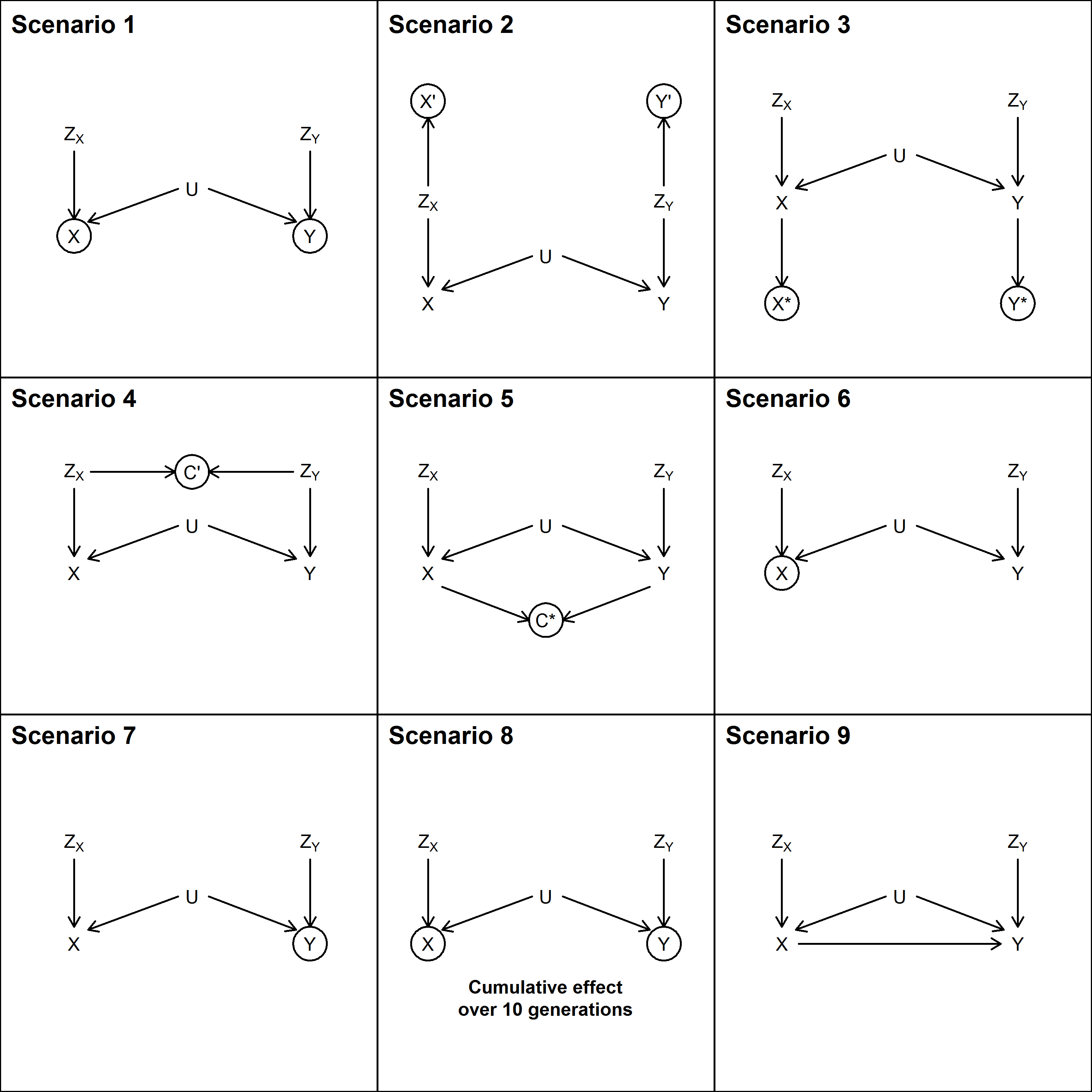

Supplement: Supplementary file 3 — Supporting Information [file GEPI-42-608-s003.tiff]

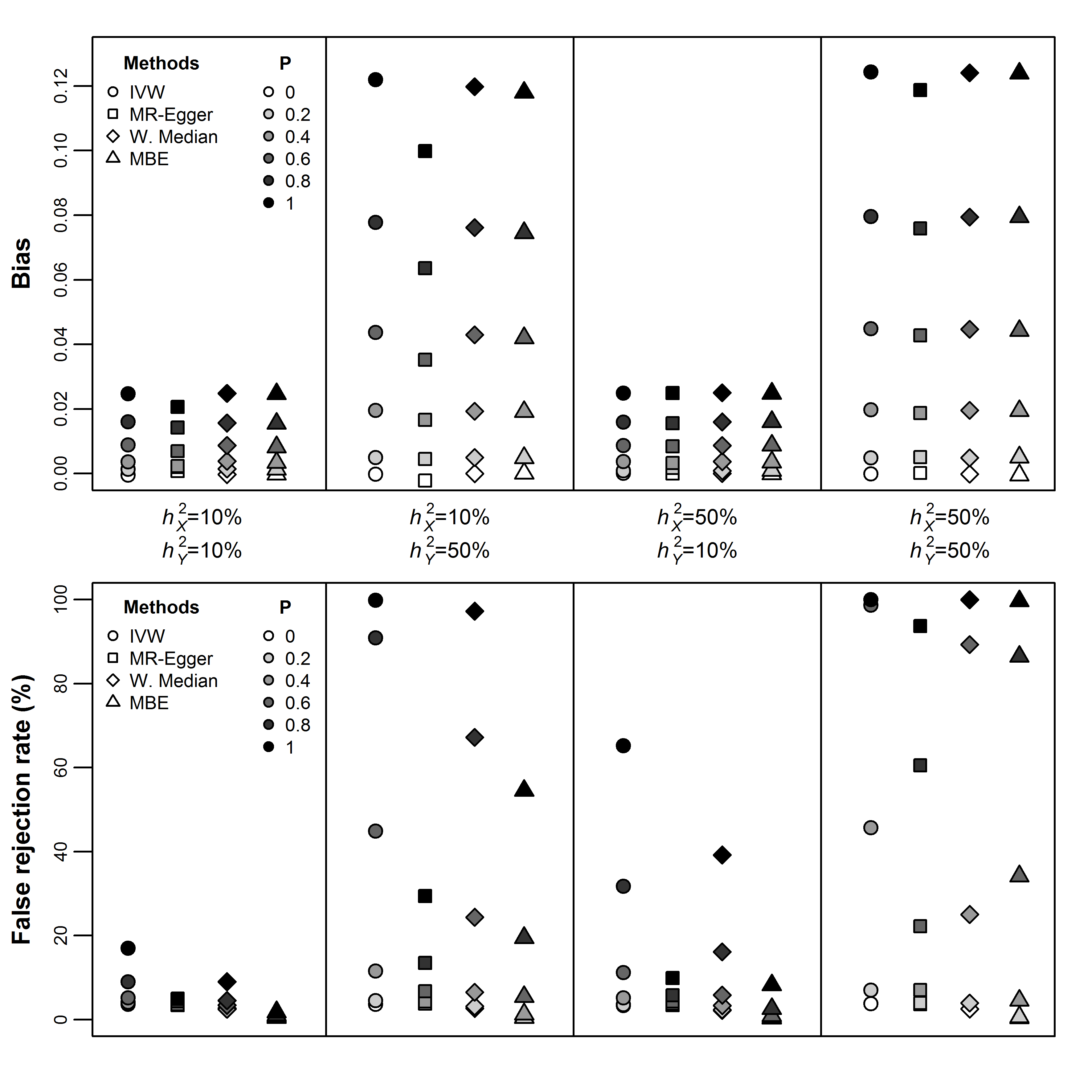

Supplement: Supplementary file 4 — Supporting Information [file GEPI-42-608-s004.tiff]
